# Multimodal nanoparticle analysis enabled by a polymer electrolyte nanopore combined with nanoimpact electrochemistry†

**DOI:** 10.1039/d4fd00143e

**Published:** 2024-07-22

**Authors:** Eugene Gyasi Agyemang, Samuel Confederat, Gayathri Mohanan, Mahnaz Azimzadeh Sani, Chalmers Chau, Dylan Charnock, Christoph Wälti, Kristina Tschulik, Martin Andrew Edwards, Paolo Actis

**Affiliations:** a Department of Chemistry and Biochemistry, University of Arkansas Fayetteville AR 72701 USA maedw@uark.edu; b Bragg Centre for Materials Research, University of Leeds LS2 9JT UK p.actis@leeds.ac.uk; c School of Electronic and Electrical Engineering and Pollard Institute, University of Leeds Leeds LS2 9JT UK; d Ruhr University Bochum Universitätsstraße 150 Bochum 44801 Germany; e Materials Science and Engineering, University of Arkansas Fayetteville AR 72701 USA

## Abstract

Nanopores are emerging as a powerful tool for the analysis and characterization of nanoparticles at the single entity level. Here, we report that a PEG-based polymer electrolyte present inside the nanopore enables the enhanced detection of nanoparticles at low ionic strength. We develop a numerical model that recapitulates the electrical response of the glass nanopore system, revealing the response to be sensitive to the position of the polymer electrolyte interface. As proof of concept, we demonstrate the multimodal analysis of a nanoparticle sample by coupling the polymer electrolyte nanopore sensor with nanoimpact electrochemistry. This combination of techniques could deliver the multiparametric analysis of nanoparticle systems complementing electrochemical reactivity data provided by nanoimpact electrochemistry with information on size, shape and surface charge provided by nanopore measurements.

## Abstract 2

We demonstrate the multimodal analysis of a nanoparticle sample by coupling a polymer electrolyte nanopore sensor with nanoimpact electrochemistry and validate the experimental results with a numerical model.

## Graphical abstract

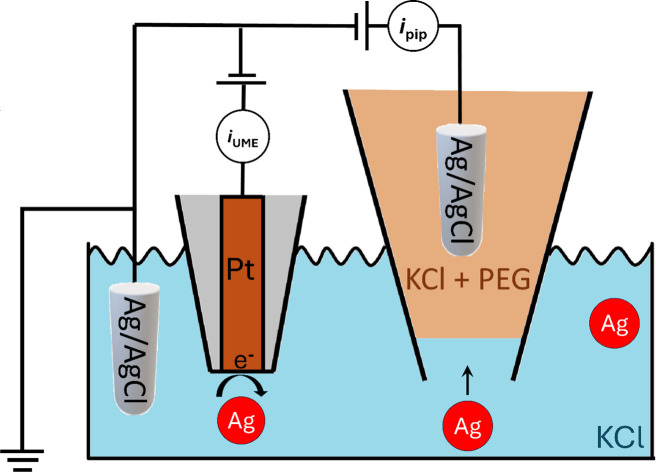

## Introduction

Over the past few decades, nanoparticles have played significant roles in scientific and technological disciplines, ranging from medicine to materials science.^1,2^ Characterizing a functional nanoparticle’s physical parameters (size, shape, *etc.*) coupled with chemical reactivity is crucial for determining structure–function relationships and to guide future developments.^3^ Moreover, the ability to characterize nanoparticles in solution and in real-time is of utmost importance as it would allow, for example, in-flow optimization of nanoparticle synthesis or characterization of dynamic processes in solution.^4^ However, physico-chemical characterization of nanoparticles in heterogenous mixtures is challenging.^5^ While dynamic light scattering (DLS) or UV-vis spectroscopy provide robust information on size distributions, they are ensemble-averaging techniques and, therefore, fall short in fully characterizing heterogenous nanoparticle mixtures.^6^ Nanoparticle tracking analysis (NTA) can analyse polydisperse nanoparticles with single-entity resolution, however the nanoparticles need to have a refractive index distinct from the surrounding medium or be modified with a fluorescent label.^7^ Electron microscopy approaches such as transmission electron microscopy (TEM) provide high-resolution characterization of individual nanoparticles but suffer from sampling bias, low throughput, require careful sample preparation, and are *ex situ*.^3^

**Fig. 1:**
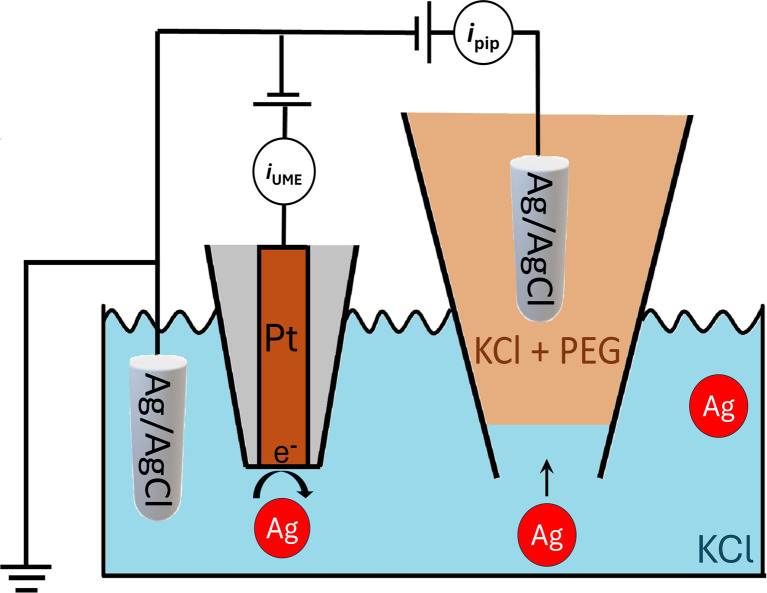
Schematic of experimental setup for nanopore sensing and nanoimpact electrochemistry.

Nanopore sensing, shown schematically in Fig. 1, is a powerful label-free electrical technique where single entities passing through a small opening between two electrolyte-filled electrode-containing reservoirs, causes temporary current modulation. In the figure, as in this work, the interior of a nanopipette is one reservoir while the orifice at the end of the pipette is the nanopore. The magnitude, duration and shape of the current modulation reflect the physical properties^8^ (*e.g.*, size, shape, charge) of the analyte and its translocation dynamics, as it is driven through the pore by an electric field and/or other forces.^9^ We have shown the large enhancement of the detection sensitivity of a conical glass nanopore with the addition of the polymer polyethylene glycol (PEG) to the electrolyte in the external bath solution.^10,11^ This discovery enabled the probing of viral RNAs,^10^ the supramolecular assembly of DNA origami,^12^ and the high-throughput characterization of heterogeneous nanoparticle mixtures at low ionic strength.^13^

Our interpretation of the mechanism of enhancement is based on evidence that the affinity of cations to PEG causes a higher anion transference number in PEG compared to aqueous solutions. This causes the ion concentration at the nanopore opening to vary with the applied potential. Ion enrichment is observed at positive potentials and ion depletion at negative potentials (measured at the internal electrode *vs.* ground in the bath). Furthermore, we postulated that the interface near the nanopore opening gets disrupted by the translocation of an analyte. We demonstrated using a range of model analytes, that such interactions could lead to alteration of the ion distribution at the tip orifice, which can result in temporary current increases.^10–12,14^

Our mechanistic description of the system suggests that ion transport in and around the interface between the polymer electrolyte and the aqueous solution, plays a key role in determining the sensitivity of the system. Herein, we investigate the reverse approach when the polymer electrolyte is placed inside the pipette and the nanoparticle samples are in the bath, a format more amenable to sensing applications.

We report that the signal enhancement is also obtained when the nanoparticle translocations are performed in a bath-to-nanopore configuration and the polymer electrolyte is only present in the nanopore, as shown in Fig. 1. We developed a numerical model that recapitulates the electrical response of the nanopore system and provides a physical explanation for the enhanced current. Furthermore, we demonstrate multimodal analysis of a nanoparticle sample by coupling nanopore sensing with nanoimpact electrochemistry. Nanoimpact electrochemistry allows for the high throughput electrochemical analyses of colloidal (sub-)micro-to nanometer sized particles based on their stochastic collisions on a micro- or nanoelectrode.^15^ This combination of techniques could allow for the multiparametric analysis of nanoparticle systems where the information on size, shape and surface charge provided by nanopore sensing can be merged with the (electro)chemical reactivity data provided by nanoimpact electrochemistry.

## Results and discussion

### Electrochemical characterization

We demonstrated that a polymer electrolyte nanopore system enables the enhanced detection of nanoparticle samples when a PEG-based polymer electrolyte is present inside the nanopipette.

We first developed a numerical model that allows determination of the coupled electric potential and ion transport within the system. The model, which is described in detail in the ESI (Section S1),† is based on that described in Marcuccio *et al.*^14^ Briefly, the nanopipette is described as a truncated cone with charged glass walls that are impermeable to ions. The bulk concentration of the ions is fixed far inside the pipette and far into the bath solution at the experimentally prepared concentration (20 mM KCl) while a potential bias, *E*, is applied between an electrode inside the pipette *vs.* ground in the bath solution. The current is calculated by integrating the fluxes of both ions across the internal electrode (eqn S3†).

**Fig. 2:**
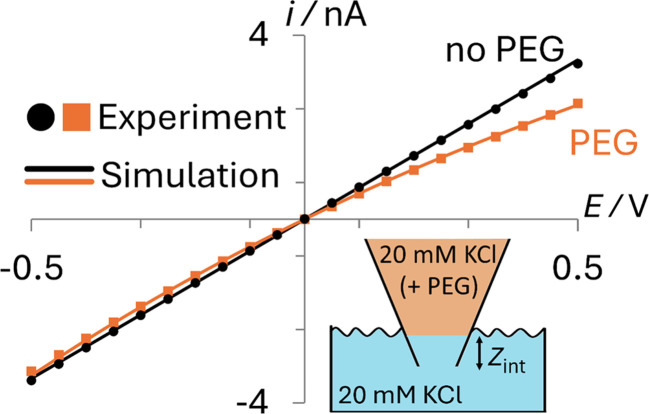
Comparison of experimental (points) and simulated (lines) *i–E* responses for PEG-containing (orange) and no PEG (black) electrolyte filled 70 nm radius nanopipettes. Bath solution: 20 mM KCl (no PEG). Pipette fill solution: 20 mM KCl with 25% (w/v) 35K-PEG. Interface between phases at *Z*_int_ = 8 μm.

The transport properties of the ions in the aqueous and PEG-containing phases, the geometry of the pipette, and the surface charge on the glass were all calculated from complementary experimental measurements, as detailed in ESI Section S2.† As can be seen in Fig. 2, simulations using these parameters (solid lines) quantitatively match experiments (points) both when the nanopipette contains 20 mM KCl (black; no PEG) or 20 mM KCl + 25% (w/v) 35K PEG (orange; PEG) that is immersed in a bath containing 20 mM KCl (Section S2.4†).

While the interface between the PEG-containing and aqueous electrolytes likely occupies a finite width mixing region, to avoid adding additional parameters to the model, we describe it as a discrete interface (see inset in Fig. 2). When the interface is modelled as residing exactly at the pipette orifice, poor agreement is observed between the experimental and simulated *i–E* response (see ESI Section S3, Fig. S6†). Yet when the interface is moved slightly inside the pipette (*Z*_int_ = 8 μm), as can be seen in Fig. 2, the simulated current (orange line) quantitatively captures the experimental *i*–*E* behavior (orange points). This apparent position of the interface likely accounts for the finite width of the interface and possibly how easily the viscous PEG-containing solution enters the nanopipette when backfilled.

**Fig. 3:**
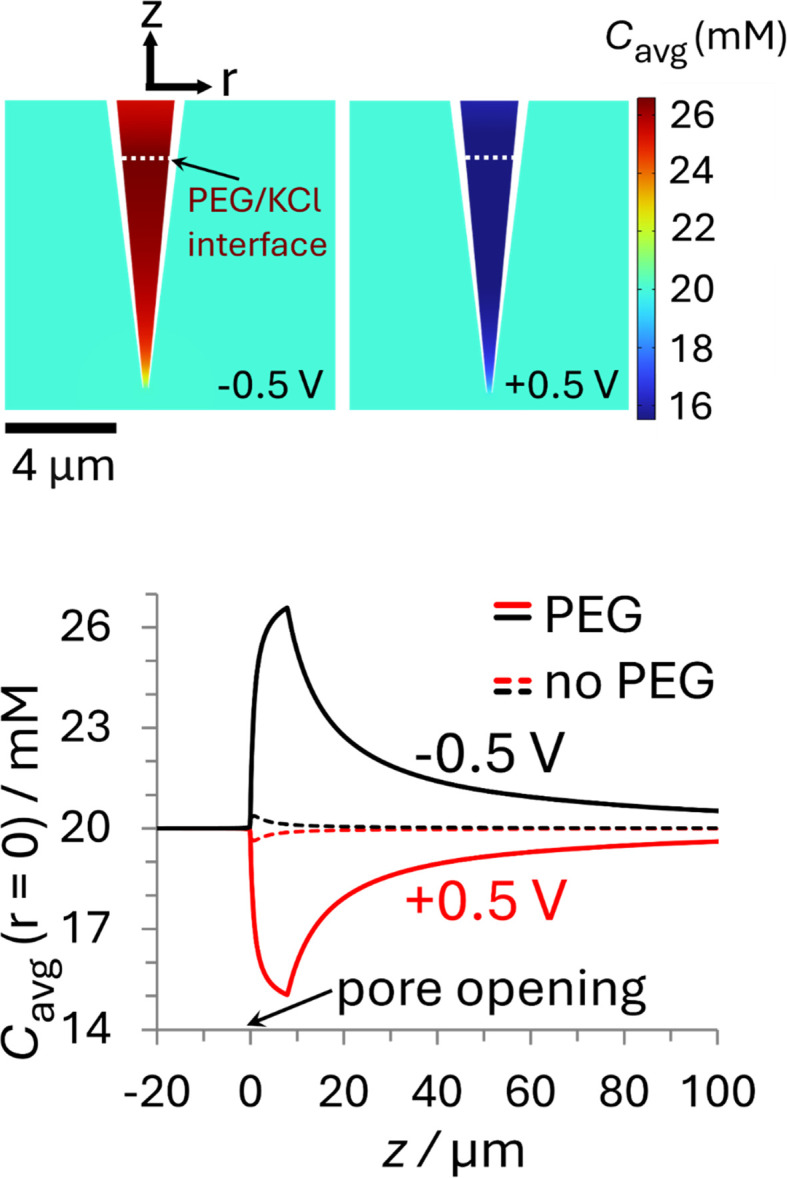
Plots of the simulated ion concentration around the nanopipette tip at ±0.5 V. (top) Color plots of average concentration (*C*_avg_ = 1/2([K^+^] + [Cl^−^])) for the PEG-in-nanopipette/KCl-in-bath configuration at (left) −0.5 V and (right) +0.5 V. The white dashed line represents the interface between phases at *Z*_int_ = 8 μm. (bottom) Average ion concentrations along the nanopipette axis of symmetry. The solid curves are for the PEG-in-nanopipette/KCl-in-bath configuration and the dashed lines for KCl in the bath and nanopipette (no PEG). Nanopipette radius: 70 nm. PEG: 25% (w/v) PEG 35K + 20 mM KCl. KCl: 20 mM KCl. The individual cation and anion concentration distributions are included in the ESI Section S5.1.† For color plots of *C*_avg_ with 20 mM KCl in the bath and nanopipette see Fig. S11.† See Fig. S10† for axial concentrations at other potentials.

The *i*–*E* response of a 20 mM PEG-electrolyte filled nanopipette immersed in a bath containing 20 mM KCl is nonlinear (rectified), with lower current magnitudes at positive potentials compared to their negative counterparts. At all potentials, the currents for the PEG-containing pipette (orange) are lower than their counterparts for the no-PEG case (black). The rectification can be understood by consideration of the ion distributions within the pipette at representative potentials of ±0.5 V, which are shown in Fig. 3 (see ESI† for concentrations at other potentials). Note, for simplicity Fig. 3 shows the average ion concentration, however this is representative of the K^+^ and Cl^−^ concentration in all but the electric double layer (see ESI Section S5.1† for details). At negative potentials (left), red and orange colors indicate that the concentration just inside the nanopipette is higher than the bulk concentration (blue-green color; 20 mM), whereas at positive potentials (right) the concentration is diminished (dark blue). These swings in concentration cause the corresponding changes in resistance that cause the rectified *i–E* response for the PEG-electrolyte filled nanopipette.

A quantitative comparison can easily be achieved by considering the axial concentration distribution along the pipette, which is shown in the lower part of Fig. 3. The solid lines, which were taken under the same conditions as the upper panel (PEG in pipette/KCl bath), but over a greater vertical range, show the concentration is enhanced up to 32% (−0.5 V) or decreased by as much as 25% (+0.5 V), with each extremum occurring at the location of the PEG/KCl interface (*z* = 8 μm). The dashed lines show the changes in concentration with no PEG in the pipette, which are due to the surface charge and geometric asymmetry of the conical nanopore.^16^ They are much smaller (±2%) relative to the PEG-in-pore configuration, but with the enhancement and depletion again occurring at negative and positive potentials, respectively (see Fig. S11 in the ESI† for colour plots for this condition). The small potential-dependent concentration variation, and hence change in conductivity, for the KCl/KCl case explains why the *i*–*E* response (black line Fig. 2) is close to linear. Interestingly, while the concentration inside the PEG-electrolyte containing nanopipette at −0.5 V is greater than at the same potential for a non-PEG containing electrolyte the diminished mobilities in PEG lead to an overall lower current.

To understand the origin of the ion accumulation/depletion within the PEG-electrolyte containing nanopipette requires consideration of the transport of the two ions in both phases. While the increased viscosity of the PEG solution leads to lower conductivity (0.119 S m^−1^*vs.* 0.386 S m^−1^ in 20 mM KCl) there are differences in the relative values of the diffusion coefficients (see ESI Section S2.4†). While the transference numbers of K^+^ and Cl^−^ in aqueous electrolyte are approximately equal (*D*_K^+^_/*D*_Cl^−^_ ≈ 0.96)^17^ the affinity of cations for the PEG leads to K^+^ having a lower transference number than Cl^−^ with 
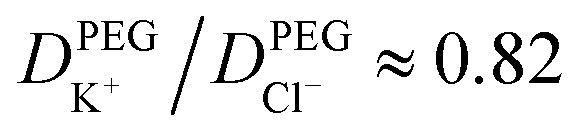
. This leads the interface between the PEG-containing and aqueous electrolytes to behave selectively for the transport of anions. At negative potentials, the anion-selective nature of the interface leads the cations that are driven inwards by the electric field to accumulate near the interface. This in turn triggers a compensatory accumulation of anions to maintain electroneutrality. At positive potentials, the opposite is true and the concentration around the end of the pipette decreases.

Ion current rectification in nanopores is typically associated with the charge on the glass surface introducing ion selectivity to a nanopore when the double layer thickness is a significant proportion of the pore diameter.^16,18^ Yet in the PEG-electrolyte system the ion selectivity arises from a fundamentally different mechanism. This can be confirmed by simulations in which the surface charge is set to 0 and which show negligible change in the rectified *i*–*E* response (see ESI Section S4, Fig. S7–S11†).

### Nanoparticle translocations

**Fig. 4:**
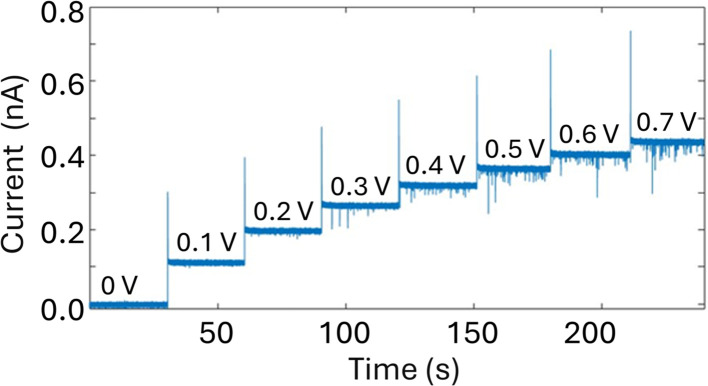
Nanopore measurements of a solution of 0.04 mg mL^−1^ 30 nm-diameter citrate Ag NPs dissolved in 20 mM KCl using a 60 nm diameter glass nanopore filled with 25% 35K PEG and 20 mM KCl with increasing applied potential. Reference electrode: Ag/AgCl frit filled with 20 mM KCl. Measurements performed with the Elements SRL nanopore reader at a sampling frequency of 100 kHz.

A glass nanopore filled with a polymer electrolyte enables the enhanced detection of nanoparticles. We first fabricated a 140 nm glass nanopore, immersed it in an electrolyte solution, and applied a potential bias between a pair of Ag/AgCl (one in the bath, one in the nanopore). The potential drives nanoparticle translocation from the trans chamber (external bath) to the cis chamber (inside the glass nanopore). Nanoparticle translocations were observed when the pore was filled with a solution of 25% 35K PEG + 20 mM KCl but not when the pore was filled with 20 mM KCl (no PEG). To improve the signal to noise ratio, we then fabricated glass nanopores with a diameter of 60 nm and probed the translocation of the 30 nm diameter silver nanosphere under a range of applied voltages (Fig. 4). Where the setup was as described for the 140 nm pore above (25% 35K PEG + 20 mM KCl in the pore). Fig. 4 shows representative ion current traces with increasing applied voltage where individual nanoparticle translocations can be identified from 200 mV.

**Fig. 5:**
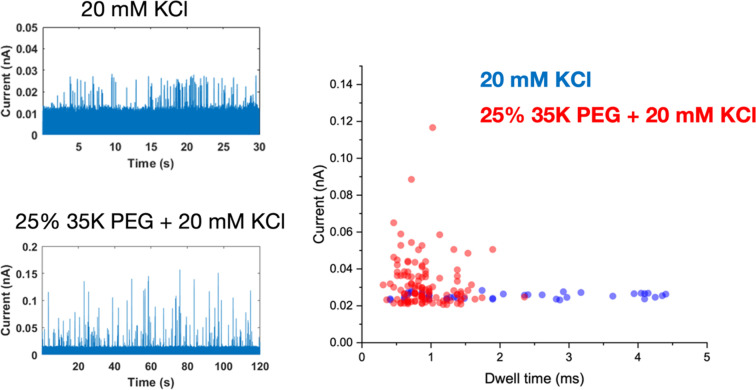
Nanopore measurements of a solution of 0.04 mg mL^−1^ 30 nm-diameter citrate-capped Ag NPs dissolved in 20 mM KCl using a 60 nm-diameter glass nanopore (a), either filled with 20 mM KCl or 25% PEG 35K and 20 mM KCl. (b) Scatter plots of the single nanoparticle translocation events measured with a glass nanopore filled with 20 mM KCl (blue dots) or 25% PEG 35K and 20 mM KCl (red dots). Reference electrode: Ag/AgCl frit filled with 20 mM KCl. Measurements performed with the Elements SRL nanopore reader at a sampling frequency of 100 kHz. Applied potential 700 mV.

Interestingly, the presence of the polymer electrolyte inside the glass nanopore enhances both the amplitude and the number of translocation events, thus facilitating nanoparticle detection (Fig. 5). We observed a similar enhancement when the polymer electrolyte was only present in the bath solution; but it has to be noted that in the “nanopore-to-bath” configuration the nanoparticle translocation led to conductive events while in the “bath-to-nanopore” we observed only resistive events. This indicates that the mechanism responsible for the signal enhancement could be different from the one reported before^14^ and its precise elucidation will require further work which is beyond the scope of this paper.

With PEG in the pipette, nanoparticle translocations can also be observed under very low ionic strength solution (5 mM KCl) and we have also demonstrated the detection of Pt nanoparticle translocation directly in a citrate buffer without the addition of any supporting electrolyte (Fig. S13†). The amplitude of the single entity events decreases with decreasing concentrations of the supporting electrolyte but, even in the absence of KCl, the signal to noise ratio is large enough to allow the robust identification of single entity events.

**Fig. 6:**
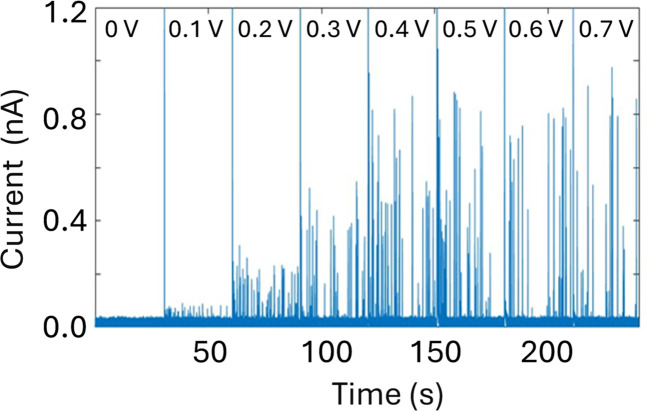
Nanoimpact measurements of a solution of 0.04 mg mL^−1^ 30 nm citrate Ag NPs dissolved in 20 mM KCL using a Pt ultramicroelectrode (10 μm in diameter) with increasing applied potential (as labelled). Reference electrode: Ag/AgCl frit filled with 20 mM KCl. Measurements performed with the Elements SRL nanopore reader at a sampling frequency of 100 kHz.

An important consequence of enabling outside-to-inside nanoparticle analysis is that multiple sensors can be integrated in the trans chamber to enable multimodal nanoparticle detection. As a proof-of-concept, we demonstrate the integration of a Pt-microelectrode within the trans chamber to perform nanoimpact measurements of the Ag nanoparticle sample. Nanoimpact experiments rely on random collision of micro or nanoparticles with a polarized electrode due to their Brownian motion in solutions, thus providing an efficient approach for electrochemical detection and characterization of electrochemically active nanoparticles. Oxidative nanoimpact events can be detected from an applied voltage of 100 mV (*vs.* Ag/AgCl) and their amplitude increases with increasing applied voltages (Fig. 6), demonstrating that the experimental setup and the buffer environment is compatible for a multi-sensor analysis of nanoparticle samples.

## Discussion

Here we reported the combination of nanopore sensing with nanoimpact electrochemistry to provide enhanced analysis of nanoparticle samples. This approach combines shape, charge, and volume sensitivity of nanopore sensing with the ability of nanoimpact electrochemistry to probe electrochemical activity. This approach uniquely enables the detection of nanoparticles under low ionic strength (20 mM) which is still a great challenge in nanopore sensing, potentially allowing the detection of nanoparticle samples prone to aggregation under high ionic strength. In the future, sensor fusion approaches^19^ could also be integrated to combine the outputs of both sensors to provide precise physical and chemical characterization of heterogenous nanoparticle samples. Importantly this integration will also require advancements in the signal processing algorithms to take full advantage of advanced machine learning routines.^20–22^

The approach described in this work involves two distinct sensors (a glass nanopore and a Pt microelectrode) to perform the multimodal nanoparticle analysis, but Pandey *et al.* have shown that an integrated nanopore-nanoelectrode setup can be implemented for single entity detection^23^ and for intracellular delivery.^24^ Similarly, Ren *et al.* constructed a nanopore field-effect transistor that could also be employed for multimodal nanoparticle sensing.^25^ An alternative approach could also rely on the application of pressure to drive the nanoparticle translocations.^26^

Also, this work complements the work by Kawaguchi *et al.*^27^ that reported the enhanced nanoparticle sensing in a highly viscous nanopore by showing that the interfacial properties at the nanopore are also responsible for its sensing performance.

The key advantage of utilising nanopipettes is that they can be integrated with micromanipulators and multi-well plates, in an a similar way to liquid handling robots, to enable the automated analysis of several analytes sequentially, in a similar way to the work of Liang *et al.*, where they employed a robotic electrochemical reader for biosensing.^28^ Also, nanopipettes can be integrated with nanomanipulators to comprise a scanning probe microscopy setup to allow for the nanoscale analysis of single entities.^29^

## Conclusion

We have shown that there is a significant enhancement of the detection sensitivity of a conical glass nanopore, for bath-to-nanopore translocating events, when the nanopore electrolyte is composed of 25% (w/v) 35K-PEG in 20 mM KCl.

We developed a numerical model that recapitulates the electrical response of the nanopore system and provides a physical explanation for the enhanced current. Our interpretation of the mechanism of enhancement is based on evidence that the affinity of cations to PEG causes a higher anion transference number in PEG compared to aqueous solutions, which generates a voltage-dependent ion concentration distribution in the vicinity of the nanopore orifice with a concentration enhancement at negative biases and depletion at positive biases. The model reveals that the electrical response of the glass nanopore is sensitive to the position of the PEG interface.

As a proof of concept, multimodal analysis of a nanoparticle sample was demonstrated by coupling the polymer electrolyte nanopore sensor with nanoimpact electrochemistry. This combination of techniques could deliver the multiparametric analysis of nanoparticle systems yielding (electro)chemical reactivity data provided by nanoimpact electrochemistry in addition to information on size, shape and surface charge provided by nanopore measurements. We hope that this approach will lead to new insights in structure–function relationships of functional nanoparticles.

## Materials and method

### Chemicals and materials

All reagents used in the translocation experiments were prepared using ultra-pure water (18.2 MΩ cm) from a Millipore system and further filtered through a 0.22 μm syringe. KCl, Triton-X, EDTA, and PEG reagents were purchased from Sigma-Aldrich. Ag and Pt spherical nanoparticles (citrate capped, 30 nm radius) were purchased from the nanoXact range from nanoComposix and were used as received. Silver wire (0.25 mm diameter) used in the nanopore experiments was obtained from Alfa Aesar.

### Electrolyte conductivity measurement

All electrolyte conductivity was measured with the Traceable™ Conductivity Meter Pen (11714226, Fisher Scientific).

### Standard nanoparticle characterization

The stability of the gold nanoparticles diluted in the KCl translocation buffer was probed by UV-vis measurements using a NanoDrop ND-1000 spectrophotometer (Thermo Scientific). The size distribution and the ζ-potential of the standard nanoparticles in pure water and 20 mM KCl solution was determined by Zetasizer NanoZS (Malvern Instruments Ltd) and are shown in Fig. S14 and 15.† All the standard nanosphere samples were used as received.

### Nanopore fabrication and characterization

The nanopores were fabricated starting from 1.0 mm × 0.5 mm quartz capillaries (QF120-90-10; Sutter Instrument, UK) with the SU-P2000 laser puller (World Precision Instruments, UK), using a two-line program: (1) HEAT, 750; FILAMENT, 4, VELOCITY, 30; DELAY, 145, PULL, 80; (2) HEAT, 600, FILAMENT, 3; VELOCITY, 40; DELAY, 135; PULL, 150. The pulling parameters are instrument specific and lead to a glass nanopore with a diameter of ≈60 nm. Adjustments of the HEAT and PULL parameters were made to fabricate other pore sizes specified in this study. The pulled glass nanopores were characterized by measuring their pore resistance in 0.1 M KCl and the pore dimensions were confirmed using scanning electron microscopy (SEM) using a Nova NanoSEM at an accelerating voltage of 3–5 kV.

### Polymer electrolyte preparation

The KCl electrolyte was first dissolved with 18.2 MΩ ddH_2_O to a final concentration of 1 M, the solution was then filtered through a 0.22 μm syringe membrane filter (E4780-1223; Starlab UK). For example, to generate 10 mL of the 50% (w/v) PEG with 20 mM KCl, 0.2 mL of the 0.22 μm filtered 1 M salt solution, 4.8 mL of 0.22 μm filtered 18.2 MΩ cm ddH_2_O and 5 g of PEG 35 kDa (ultrapure grade, Sigma-Aldrich) were mixed inside a tube. The tube was then left inside a 70 °C incubator for 2 hours and kept at 37 °C overnight. The tubes were then left on a bench for 4 hours to reach room temperature prior to use. The polymer electrolyte was then stored at room temperature.

### Nanopore translocation measurements

A Ag/AgCl wire (0.25 mm diameter, GoodFellow UK) was inserted in the glass nanopore barrel and acted as the working electrode. The counter/reference electrode consisted of a second Ag/AgCl wire electrode directly placed in the bath or, where stated in the caption, a Ag/AgCl electrode in a 20 mM KCl filled capillary separated by a glass frit. In some experiments, a Ag/AgCl frit filled with 20 mM KCl was used. The nanoparticles were driven from the external bath into the nanopipette by applying a positive potential to the working electrode placed inside the glass nanopore with respect to the reference electrode in the bath. The ion current was recorded either with a MultiClamp 700B patch-clamp amplifier (Molecular Devices) in voltage-clamp mode at a 100 kHz sampling rate with a 20 kHz low-pass filter using the pClamp10 software (Molecular Devices) or using the Nanopore reader (Elements srl) 100 kHz sampling rate with a 20 kHz low-pass filter.

### Nanoimpact measurements

Nanoimpact electrochemistry experiments were performed using a 2-electrode configuration using a Pt microelectrode (10 μm in diameter) as the working electrode and a Ag/AgCl frit filled with 20 mM KCl as the reference/counter electrode. Data were acquired with the nanopore reader (Elements srl) with a 100 kHz sampling rate and a 20 kHz low-pass filter.

### Numerical simulations

A numerical model was developed which included equations describing the electrical potential and ion concentrations distribution inside and around the glass nanopore filled with polymer electrolyte. The pore geometry was approximated as a truncated cone. The simulations were implemented using the commercial finite element software COMSOL Multiphysics (version 6.0 & Chemical Reaction Engineering module) to solve the coupled Poisson and Nernst–Planck equations (see ESI Section S1.1†). The simulations are based on our model of a glass nanopore immersed in a polymer electrolyte^14^ and are described briefly below. Further details, including the determination of physical parameters, can be found in Section S1.1 of the ESI.†

Boundary conditions, which are listed in the ESI†, were chosen to reflect the experimental system, and include an applied potential at the internal electrode *vs.* an external electrode at ground, surface charge on, and no ion transport through, the glass walls, and bulk solution concentrations. Ion transport depends on the phase (PEG + KCl or KCl) with diffusion coefficients chosen to match the experimentally measured solution conductivities. In KCl, the diffusion coefficients of the K^+^ and Cl^−^ are approximately equal^17^ (*D*_K^+^_ : *D*_Cl^−^_ = 0.49 : 0.51) while in the PEG Cl^−^ is more mobile^14^ with the ratio *D*_K^+^_ : *D*_Cl^−^_ = 0.45 : 0.55 (see ESI† for details). For simplicity, we took the interface between the PEG + KCl and KCl to have zero width, *i.e.*, mixing of the solutions was neglected, with the interface determined to reside inside the pipette by ∼8 μm (see ESI Section S3† for details).

## Data availability

The data that support the findings of this study are openly available from the University of Leeds data repository at https://doi.org/10.5518/1599.

## Conflicts of interest

The authors declare no competing interests.

## Supplementary Material

FD-257-D4FD00143E-s001

FD-257-D4FD00143E-s002
