# Supplementary material for: Multimodal nanoparticle analysis enabled by a polymer electrolyte nanopore combined with nanoimpact electrochemistry
Source: Faraday Discuss. 2024 Jul 22;257:303–15. doi: 10.1039/d4fd00143e (PMC11563348; doi:10.1039/d4fd00143e)
Supplement: FD-257-D4FD00143E-s001 [file FD-257-D4FD00143E-s001.pdf]

Supplementary Information (SI) for Faraday Discussions.
This journal is © The Royal Society of Chemistry 2024

Supporting Information for:

Multimodal nanoparticle analysis enabled by a polymer electrolyte nanopore combined with nanoimpact electrochemistry

Eugene Gyasi Agyemang,[a,b,#] Samuel Confederat,[c,d,#] Gayathri Mohanan,[c,d,#] Manhaz Azimzadeh Sani,[e] Chalmers Chau,[c,d] Dylan Charnock,[c,d] Christoph Wälti,[c,d] Kristina Tschulik,[e] Martin Andrew Edwards[a,b*] and Paolo Actis[c,d*]

[a] Department of Chemistry and Biochemistry, University of Arkansas, Fayetteville, AR, 72701, USA

[b] Materials Science and Engineering, University of Arkansas, Fayetteville, AR, 72701, USA

[c] Bragg Centre for Materials Research, University of Leeds, LS2 9JT, UK

[d] School of Electronic and Electrical Engineering and Pollard Institute, University of Leeds, Leeds LS2 9JT, UK

[e] Ruhr University Bochum, Universitätsstraße 150, 44801 Bochum, Germany

# These authors contributed equally to the work

* Corresponding authors; maedw@uark.edu; p.actis@leeds.ac.uk

Table of Contents

S1. Description of Simulations S4

S1.1. Physics used for finite-element modelling S4

S1.1. Boundary conditions and physical parameters S6

S1.2. Mesh S8

S1.3. Ion current measurement S9

S2. Parameters for Numerical Simulations S10

S2.1. Nanopore Radius S10

S2.2. Estimating the Inner Half Cone Angle S10

S2.3. Diffusion Coefficients in KCl S11

S2.4. Estimating Surface Charge Density on Glass Nanopipette Inner Walls S12

S2.5. Diffusion Coefficients in PEG containing solutions S13

S3. Effect of Position of the KCl + PEG/KCl Interface S15

S4. Influence of Surface Charge S16

S5. Supplementary Simulated Electric Potential and Concentration Distributions S17

S5.1. K^+ and Cl^- concentrations along the symmetry axis S17

S5.2. Electric Potential distribution S18

S5.3. Average ion concentrations along the symmetry axis under different applied potentials (PEG in nanopipette/KCl in bath) S18

S5.4. Average ion concentration profiles for KCl in nanopipette and external bath S20

S6. Detection of Ag nanoparticles with 70 nm radius nanopore S21

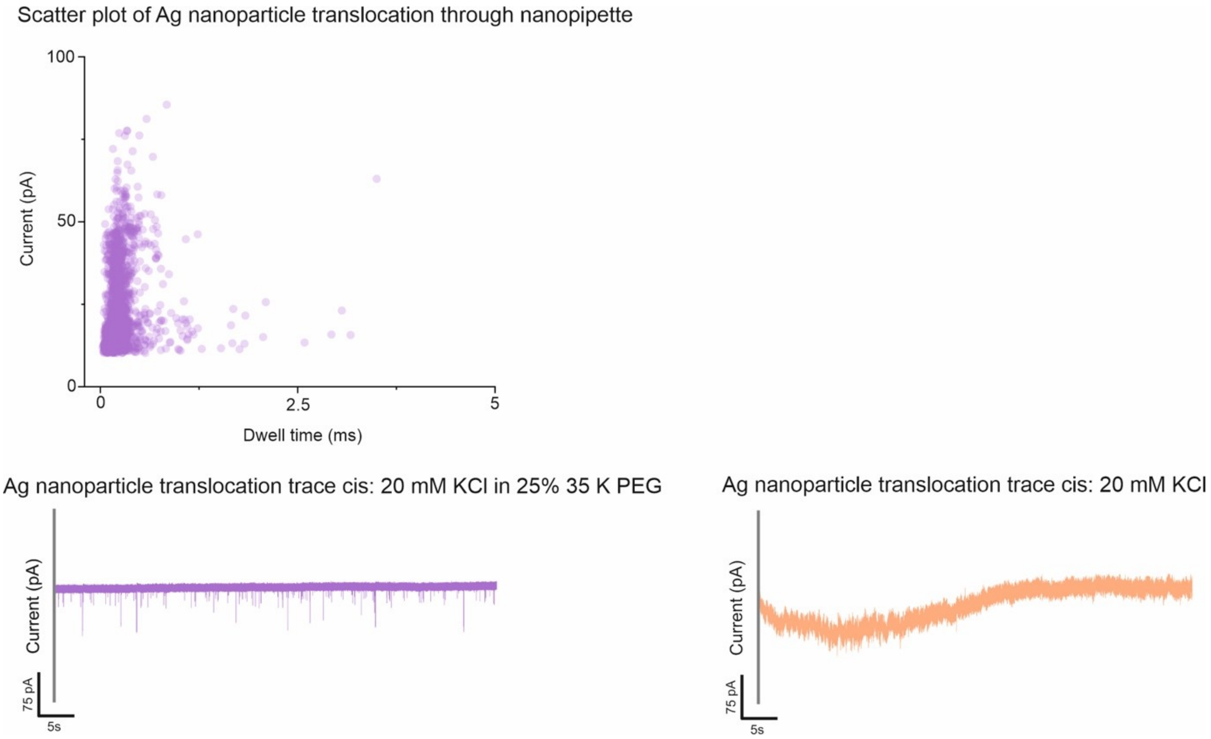

21

Figure S12. Scatter plot and ion current traces of Ag nanoparticles translocation using 70 nm radius nanopipette. 21

S7. Detection of Pt nanoparticles under different ionic strength 22

S8. Confirmation of stability of nanoparticles in 20 mM KCl 23

S9. References 24

S1. Description of Simulations

Simulations were developed that describe the electric field and ion distribution within the electrolyte-filled nanopipette and bath. These simulations broadly follow those described in Marcuccio et al. 2023 [1] and account for the different transport properties in different phases (PEG-containing and PEG-free KCl solutions), but differ in the locations of the phases. The physical parameters for the simulations (pipette geometry, surface charge and diffusion coefficients of the ions in the two phases, etc.) were taken from literature or determined through independent experiments, following the procedures described in Marcuccio et al. 2023 [1]. The processes used to obtain each of the parameters for the numerical simulations are described in section S6 of this document.

A detailed description of the physics included in the simulations is provided below. Additional details including solver parameters and details of the meshing used is provided through the inclusion of a COMSOL model report, which is provided as a separate supporting information document.

S1.1. Physics used for finite-element modelling

The concentration $c_i(r, z)$ for species i (=K^+/Cl^-) and the potential $\phi(r, z)$ were determined by solving the coupled Nernst-Planck and Poisson equations in the 2D-axisymmetric geometry illustrated in Figure S1. Ionic flux in the simulation domain, $\vec{J}_i$, was modelled with the Nernst-Planck equation (Eq. S1), which was solved in the steady-state conditions ($\nabla \cdot J_i = 0$).

$$\vec{J}_i = - D_i^{\alpha} \nabla c_i + \frac{z_i F}{RT} D_i^{\alpha} c_i \nabla \phi \qquad \text{Eq. S1}$$

where D_i^α is diffusion coefficient of species *i* in phase α (= PEG or KCl, referring to 20 mM KCl + 25% 35K PEG and 20 mM KCl, respectively) and z_i its ion valence number. *F*, *R*, and *T* take their usual meanings.

The relationship between the electric potential and ion concentrations was described using the Poisson equation:

$$\nabla^2\phi = -\frac{F}{\varepsilon^\alpha}\sum_i z_i c_i \qquad \text{Eq. S2}$$

where ε^α is the electric permittivity in domain α.

By modelling the ionic flux with the Nernst-Planck equation we implicitly assume negligible effects from convective flow, e.g., due to electroosmosis. Previous measurements from a PEG-in-bath configuration [1] and favourable comparison of our simulated *i-E* behaviour to experiment (*vide infra*) both suggest that this is a reasonable simplification to make for this experimental system.

To aid in efficient numerical convergence of simulations, initial conditions were chosen that considered the electric double layer (assuming a planar interface) and a potential drop in the pipette (assuming uniform concentration). Analytical expressions of the Gouy-Chapman equation [2] are combined with those for the potential drop in a pipette (expressions akin to those described by Wei et al. [3] and Edwards et al. [4]). Full details including these equations and their implementation are given in the COMSOL model report, which is available as a separate Supplementary Information file.

S1.1. Boundary conditions and physical parameters

Boundary conditions were chosen to describe the physics of the experimental system and are listed in **Table S1** in which the boundary numbers associate with those given in **Figure S1**. In these definitions, $\boldsymbol{n}$ is the inward-pointing unit normal vector, and σ is the surface charge on the glass. Boundary 1 represents the surface of the Ag/AgCl quasi-reference counter electrode in the nanopipette where a voltage bias, E_{app}, was applied relative to the ground on boundary 3, which is representative of the surface of the ground electrode (Ag/AgCl) in the bath. Boundary 2 is quartz glass walls with a surface charge density of σ = -2 mC/m^2 while boundary 4 represents the axis of symmetry.

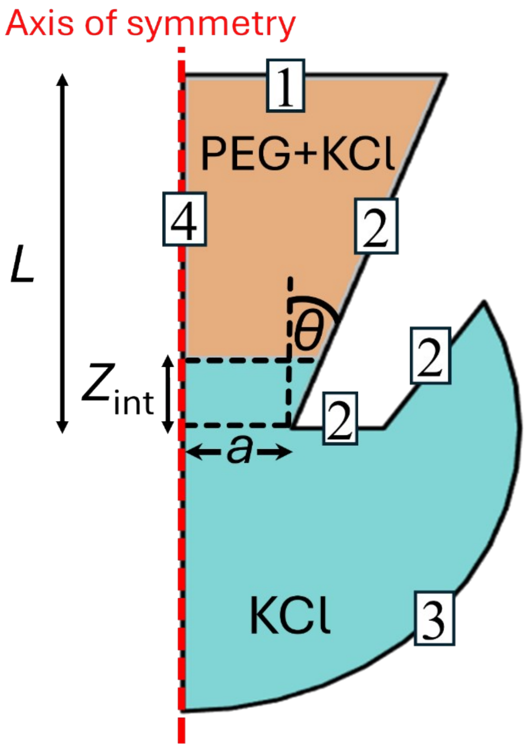

Figure S1. 2D-axisymmetric geometry of a conical glass nanopipette immersed in an electrolyte solution, as used for finite element simulations (not to scale). Boundary numbers and key geometric parameters are labelled (pore tip radius (a), inner half-cone angle (θ), PEG/noPEG interface position (Z_{int}), and pore length (L)). Note, phases are labelled for the PEG-in-nanopore configuration, but other configurations were also simulated as detailed in the text.

Table S1. Boundary conditions applied in finite-element model.

BOUNDARY	NERNST-PLANCK	POISSON
Pipette solution (1)	c_i = 20 mM*	ϕ = E_{app}*
Nanopipette walls (2)	$n \cdot \vec{J}_i$ = 0	σ = $n \cdot \varepsilon^{\alpha} \nabla \phi$
Bath solution (3)	c_i = 20 mM*	ϕ = 0 V*
Axis of symmetry (4)	Axial symmetry ($\boldsymbol{n}\vec{J}_i$ = 0)	Axial symmetry ($n \cdot \nabla\phi = 0$)

*Within the electric double layer there is a small deviation in these boundary conditions where the values were adjusted based on the Gouy-Chapman theory. I.e., near the intersection of boundaries 1 & 2 and 2 & 3 meet, the cation (anion) concentration on boundaries 1 & 3 is increased (decreased) and the electric potential increased in a distant-dependent manner to account for the negative surface charge. This treatment leads to consistency in boundary conditions and superior numerical stability. Full details of this treatment are provided in the COMSOL model report, which is available as an additional supporting information file.

The physical parameters used in the simulations are listed in **Table S2** and were determined from complementary experiments as referred to in the *Source* column and described in detail in the Supporting Information section S6.

Table S2. Values of physical parameter used in finite-element model and references to how they were determined. PEG = 20 mM KCl in 25% w/v 35K PEG, KCl = 20 mM KCl.

PARAMETER	VALUE	SOURCE
D_{Cl-}^{KCl};D_{K+}^{KCl} (m^2 / s)	2.58×10^{-9}; 2.48×10^{-9}	Best fit to expt. (S6.3)
D_{Cl-}^{PEG};D_{K+}^{PEG}(m^2 / s)	8.57×10^{-10}; 7.01×10^{-10}	Best fit to expt. (0)
κ^{α} (S / m)	KCl = 0.386, PEG = 0.119	Measured
ε^{α}	KCl = 80, PEG = 52	Literature values[5, 6]
Pore radius (nm)	71	Measured (Electron microscopy) (S6.1)
Surface Charge on glass (mC/m^2)	-2	Best fit to expt. (0)
pore half-angle	4.9°	Determined from *i-E* response (S6.2)

S1.2. Mesh

Figure S2 illustrates the mesh used for finite element simulations. The complete mesh consists of 33491 domain elements and 1838 boundary elements. Boundary element meshing with a width of 1/10 of the Debye length was used on the glass (Boundary 2) to efficiently capture the electric double layer. The average mesh element quality was 95.6%. The mesh was determined to be sufficiently fine as simulations with a finer mesh did not materially alter the measured currents or concentrations (data not shown). Full details of the mesh are available in the COMSOL model report which is uploaded as a separate supporting information file.

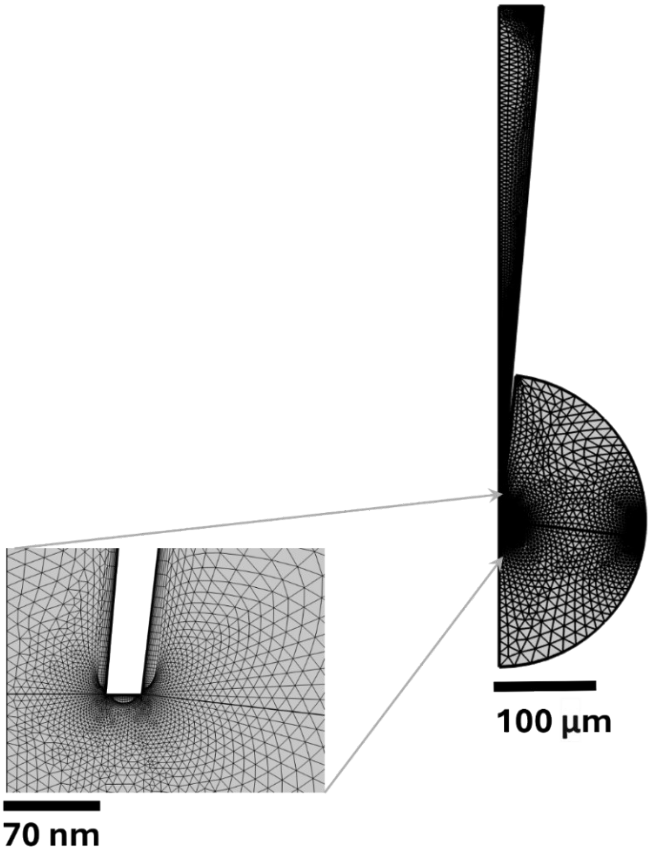

Figure S2. Mesh used for finite element simulations. (Inset) Zoomed-in view of mesh at the nanopipette tip aperture showing the boundary layer mesh used on the glass surface.

S1.3. Ion current measurement

The ion current was calculated by integrating the total ion flux through the top boundary 1 (inner electrode, see **Figure S1**) as shown:

$$i = 2\pi F \int_{bound.\ 1} r(\vec{J}_{K+} - \vec{J}_{Cl-}) \cdot n \, dr$$

Eq. S3

where *r* is the radial coordinate and the 2π*r* accounts for the integral of rotation.

S2. Parameters for Numerical Simulations

The parameters used in numerical simulations described in section S1 were determined from a series of complementary experiments, application of theoretical results, and literature values. Below, we describe in detail how the parameters were determined. The process closely mimics that used in Marcuccio et al. 2023.[1]

S2.1. Nanopore Radius

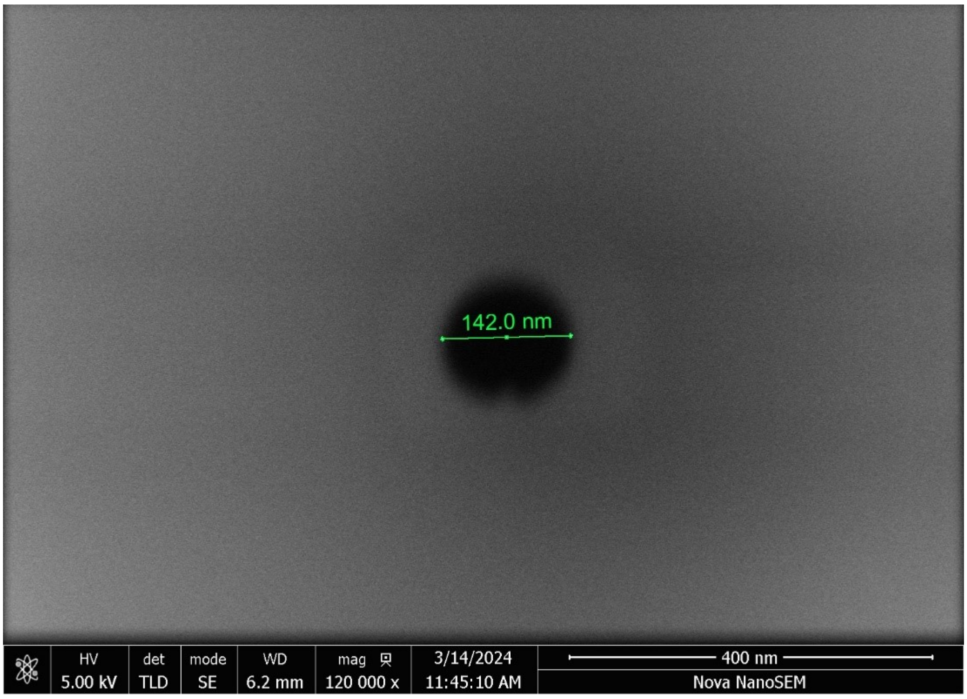

Figure S3. Scanning electron micrograph of a representative 70-nm radius nanopipette used in this work.

S2.2. Estimating the Inner Half Cone Angle

With negligible surface charge and/or sufficiently high ionic strengths electrolyte, conical nanopores produce *i-E* responses that are essentially ohmic, from which the resistance, R_p, can be calculated. The *i-E* response of a nanopipette filled with 20 mM KCl in a bath containing 20 mM KCl is shown as red points in Figure S4.

The resistance of a conical pipette in a uniform solution is described by equation Eq. S4 [7, 8].

$$R_p = \frac{L}{\pi \kappa r\ (a\ L\ tan(\theta))} + \frac{1}{4\kappa a} \qquad \text{Eq. S4}$$

where a is the tip aperture radius, L the length of the pore, κ the electrical conductivity of the electrolyte, ϑ the inner half-cone angle. For sufficiently long pipettes (L tan(θ) ≫ a), this becomes:

$$R_p = \frac{1}{\kappa a}\left(\frac{1}{\pi\ tan(\theta)} + \frac{1}{4}\right) \qquad \text{Eq. S5}$$

Using the measured pore resistance, the pore radius, $a = 71\ \mathrm{nm}$, estimated from scanning electron microscopy (see Section S6.1 for details), the measured solution conductivity, κ(20 mM KCl) = 0.386 S/m, and rearranging Equation Eq. S5 we get that the inner half-cone angle $\vartheta \approx 4.9°$.

S2.3. Diffusion Coefficients in KCl

The bulk solution conductivity in phase α (κ^{α}) can be related to the diffusion coefficients for each species via the Nernst-Einstein equation. For the symmetric KCl electrolyte, this gives:

$$\kappa^{\alpha} = \frac{(D_{Cl^-}^{\alpha} + D_{K^+}^{\alpha})}{RT} F^2 c_b \qquad \text{Eq. S6}$$

where c_b (= 20 mM) is the bulk concentration.

In dilute aqueous solution (T = 25 °C), the bulk diffusion coefficients of K^+ and Cl^-, are reported as 1.957 × 10^{-9} m^2/s and 2.032 × 10^{-9} m^2/s, respectively [9]. Assuming that the ratio of diffusion coefficients is maintained in our experimental conditions (20 mM aqueous solution, ~20 °C) the experimentally measured conductivity, κ(20 mM KCl) = 0.386 S/m, gives that $D_{K+}^{KCl} =$ 2.48×10^{-9} m^2/s and $D_{Cl-}^{KCl} =$ 2.58×10^{-9} m^2/s.

S2.4. Estimating Surface Charge Density on Glass Nanopipette Inner Walls

The surface charge on the glass, σ, induces a rectification in the *i-E* response [10, 11]. The value of σ was determined through the best fit of simulations (lines) to the experimentally measured *i-E* response for a 20 mM KCl-filled pipette in a 20 mM KCl bath (points) as shown in Figure S4. The simulations use values of *a* and θ as determined in sections S6.1 and S6.2, respectively and diffusion coefficients D_{K+}^{KCl} and D_{Cl-}^{KCl} as determined in section S6.3 while σ was varied. The results of simulations with different values of σ are shown as solid lines in Fig S4.

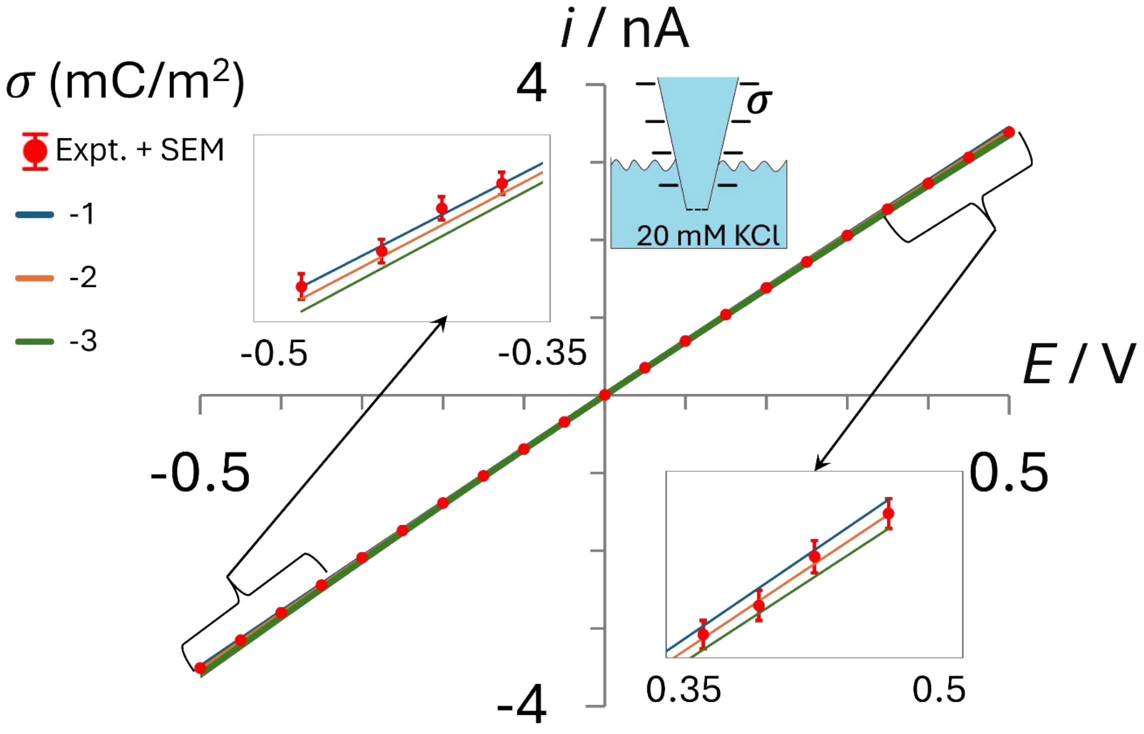

Figure S4. Comparison of experimental and simulated *i-E* responses for a pipette containing 20 mM of KCl in a bath containing the same electrolyte (no PEG present in either solution). The surface charge on the nanopipette walls, *σ*, was varied in the simulations to determine its value. The nanopore has a radius of 70 nm, half cone angle of 4.9°, and κ^{KCl} = 0.386 S/m.

S2.5. Diffusion Coefficients in PEG containing solutions

PEG has an affinity for cations, this decreases the fractional contribution of the cations to the conductivity compared to an aqueous solution where the K^+ and Cl^- have approximately equal contributions [12]. As no literature data on the ion diffusion coefficients in 35K-PEG were available, we determined their values through a combination of bulk conductivity measurements and the simulated and experimental nanopipette voltammetry.

The bulk conductivity κ (20 mM KCl + 25% w/v 35K-PEG) was measured at 0.119 S/m (see Materials and methods section in the main text for details) and is related to the two diffusion coefficients as described by equation Eq. S6. This measurement reduces the number of unknowns from two to one, which we express as the relative conductivity of the two ions ($D_{K+}^{PEG}/D_{Cl-}^{PEG}$). To determine this ratio we performed nanopipette voltammetry using the PEG-in-bath configuration, which we previously showed to be highly sensitive to the relative diffusion coefficients of the two species in the PEG-containing phase [1].

The red points in **Figure S5** show the nanopipette voltammetry with a 20 mM KCl-filled nanopipette immersed in a bath containing 25% w/v 35K-PEG + 20 mM KCl. Simulations of this situation, which are shown as solid lines, vary $D_{K+}^{PEG}/D_{Cl-}^{PEG}$ while maintaining the bulk conductivity κ(20 mM KCl + 25% w/v 35K-PEG) = 0.119 S/m, as described by equation Eq. S6. Values of D_{K+}^{KCl} and D_{Cl-}^{KCl}, surface charge and pipette geometry used the values determined above (see sections S6.3, 0, S6.1 & S6.2, respectively). The best match to experiment was found at $D_{K+}^{PEG}/D_{Cl-}^{PEG} = 0.82$ which corresponds to K^+ contributing 45% of the ion conductivity and Cl^- the remaining 55%. This gives $D_{K+}^{PEG} =$ 7.01×10^{-10} m^2/s and $D_{Cl-}^{PEG} =$ 8.57×10^{-10} m^2/s.

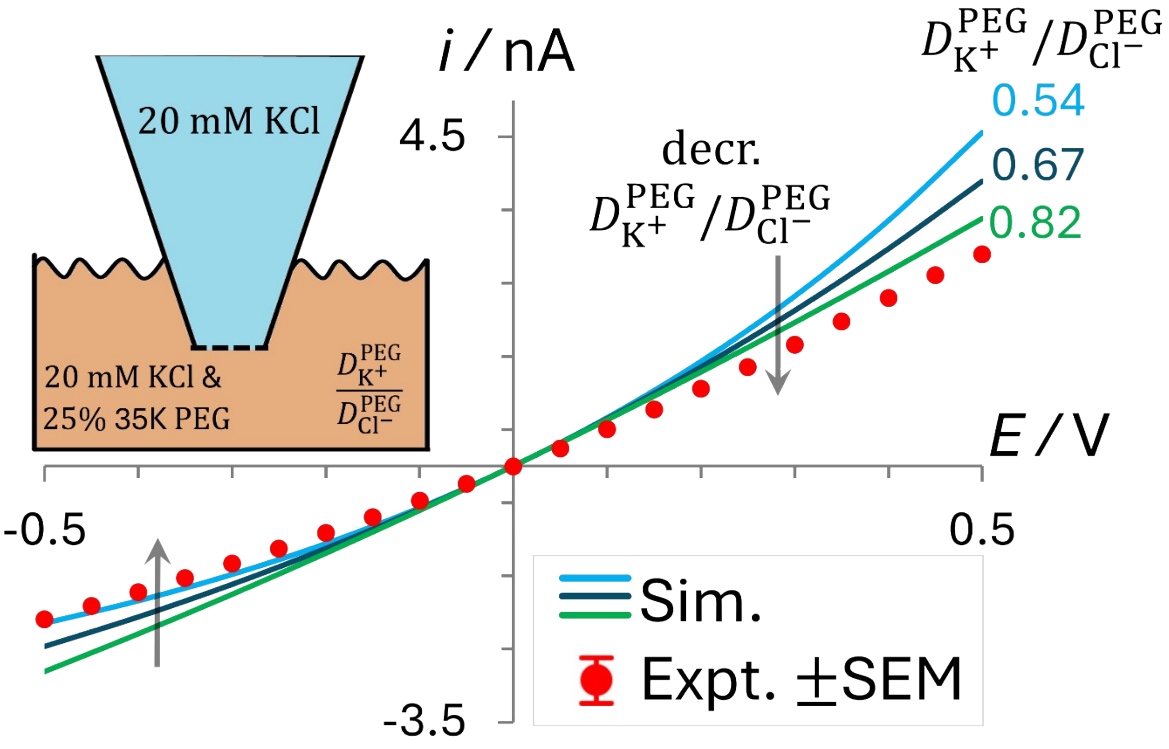

Figure S5. Comparison of simulated *i-E* responses for a PEG-in-bath/KCl-in-pipette configuration with different ratios of the diffusion coefficient of K^+ and Cl^- in the PEG-electrolyte phase. In the experiment, the green curve ($D_{K+}^{PEG}/D_{Cl-}^{PEG} =$ 0.82) was found to be the best fit to the experiment *i-E* response. The nanopore has a radius of 70 nm and a half cone angle of 4.9°. External bath solution: 25% w/v PEG 35K + 20 mM KCl. Pipette fill solution: 20 mM KCl.

S3. Effect of Position of the KCl + PEG/KCl Interface

The simulated *i*-*E* response with the KCl + PEG/KCl interface at the pore opening (golden line, **Figure S6**), which uses parameters determined from complementary experiments, fits poorly to the experimentally measured response in the same conditions (red points). This suggests that the location of the interface, which we simulate as a discrete location, is instead somewhat inside the pipette. This hypothesis is supported by simulations where the distance of the interface from the pore orifice, Z_{int}, was varied. Higher currents with increased rectification are seen with increasing Z_{int} and a near-perfect match to the experiment is observed at Z_{int} = 8 µm, the red solid line in Figure S6 (values of Z_{int} from 6 µm to 10 µm in the model show a considerably good fit to the experiment). The location of the discrete interface in the simulations may represent the approximate midpoint of a true (diffuse) interface in the experimental measurements.

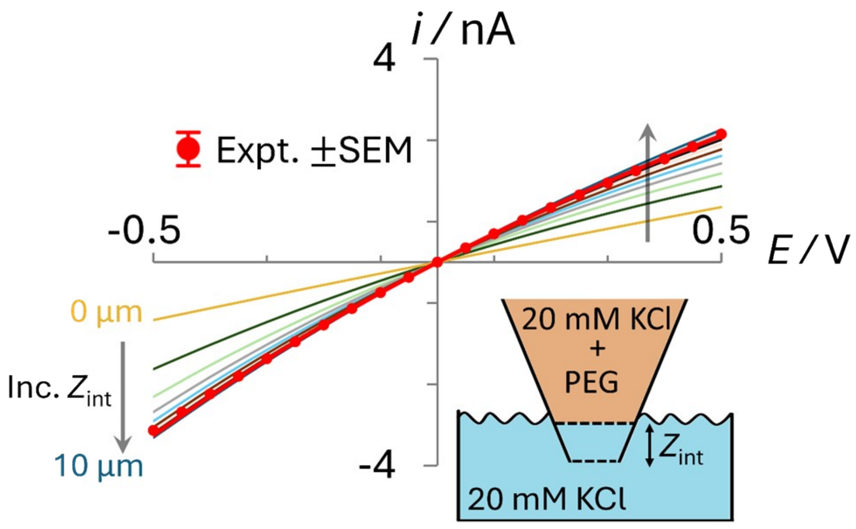

Figure S6. Experimental (red dots) and simulated (curves) *i*-*E* responses for PEG-in-nanopipette / KCl-in-bath configuration in which the interface location, Z_{int} (distance from the pore orifice), varies between 0 µm and 10 µm (1 µm intervals). Bath solution: 20 mM KCl (no PEG). Pipette fill solution: 20 mM KCl with 25% w/v 35K-PEG. Error bars representing the standard error of the mean of the experimental points (*N* = 3) are within the size of the data points.

S4. Influence of Surface Charge

It has previously been reported that with appropriate experimental conditions, the negative surface charge on the glass nanopipette wall can result in negative rectification of ionic current when the pipette and bath have identical composition [10, 11, 13]. However, while rectification is also observed with the PEG-in-nanopipette configuration, the contribution of surface charge to the observed rectification in this configuration is minimal. This can be seen in **Figure S7** which shows simulated *i-E* curves for the PEG-in-Nanopipette / KCl-in-Bath configuration with the surface charge set to 0 (black line) or the experimentally determined value (-2 mC/m^2); see section 0 for details of surface charge determination. The minimal difference in the *i-E* responses indicates that the observed rectification, and by corollary the observed signal enhancement, is dominated by the interaction of the PEG with the K$^+$ ion.

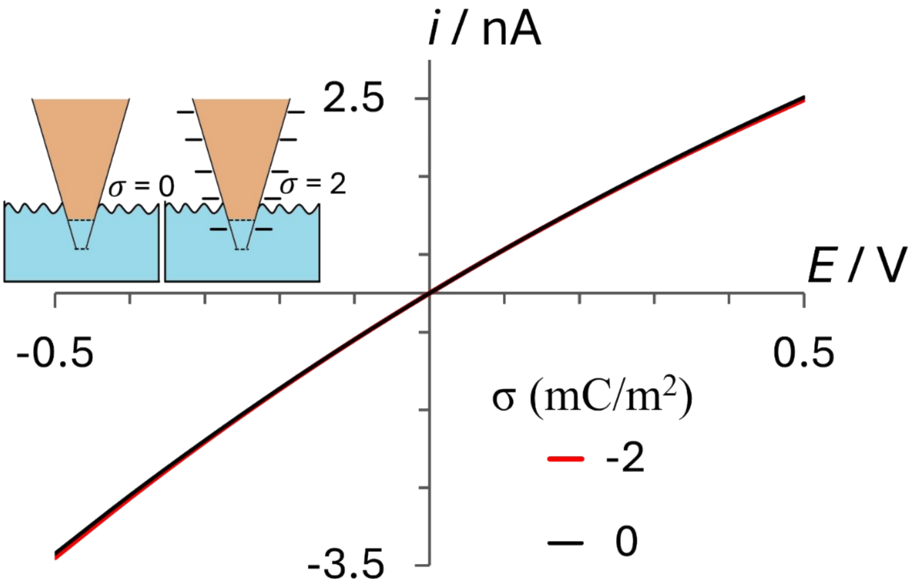

Figure S7. Simulated *i-E* responses for a nanopipette containing 20 mM KCl 25% w/v 35K-PEG in a 20 mM KCl external bath (no PEG) without (black curve) and with (σ = -2 mC/m^2, red curve) surface charge on the walls. The PEG electrolyte interface was set to a Z_{int} = 8 µm.

S5. Supplementary Simulated Electric Potential and Concentration Distributions

The distributions of the electric potential and ion concentrations within the pore provide insight into the behaviour of the nanopore, as discussed in the main text. The additional plots provided below support those shown in the main text.

S5.1. K^+ and Cl^- concentrations along the symmetry axis

Figure 2 of the main text shows the average concentration $(1/2([K^+] + [Cl^-]))$ where it was claimed that this was representative of the concentrations of either species. This claim is supported by the plot of the individual ion concentration along the nanopore axis of symmetry shown in **Figure S8**. The identical concentration profiles indicate electroneutrality is respected, which is true throughout the simulation domain except for the electric double layer, vindicating the use of average concentrations in the main text.

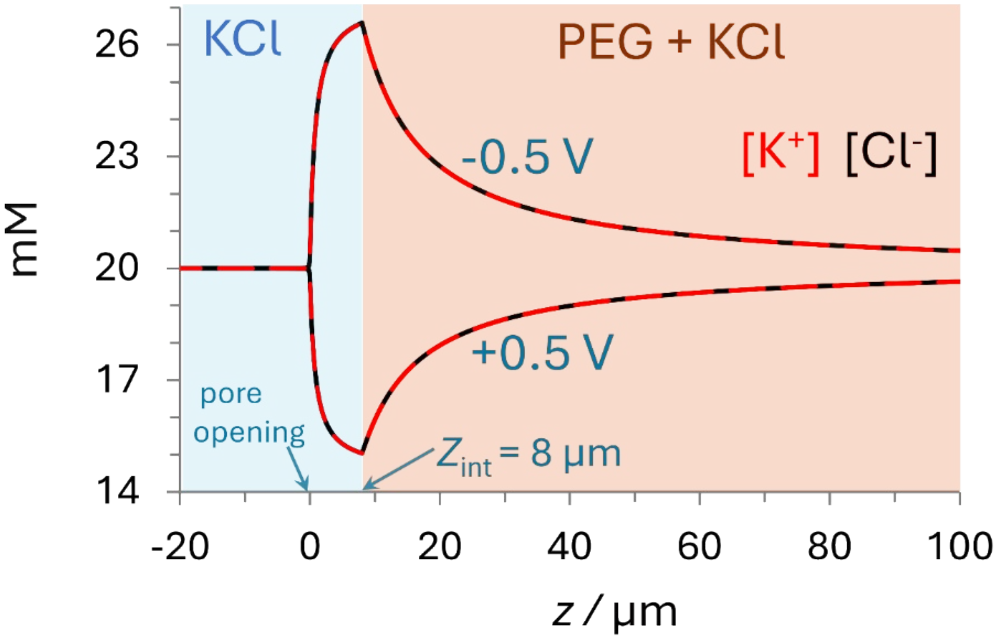

Figure S8. Simulated K^+ (red dashed lines) and Cl^- (black solid line) concentration profiles along the nanopore axis of symmetry (red dashed line in **Figure S1**) for $\pm$ 0.5 V. A 70-nm-radius nanopipette was filled with 25% w/v PEG 35K + 20 mM KCl solution and the bath solution has 20 mM KCl.

S5.2. Electric Potential distribution

As expected, all the potential drop occurs near the nanopipette orifice (z = 0 µm), where the resistance is largest. **Figure S9** illustrates this using surface colour plots and axial profiles for biases of +0.5 and -0.5 V. In these simulations 25% w/v PEG-35K in 20 mM KCl is in the nanopipette and 20 mM KCl in the external bath. Interestingly the axial potential profile captures the difference in the resistance within the PEG phase and the KCl phase that occupies the remainder of the nanopore, separated by an interfacial barrier located at Z_{int} = 8 µm: the largest potential drop occurs within the KCl region within the pore (~80% of the potential drop) and the remainder of the potential is dropped within the KCl+PEG-filled nanopore region.

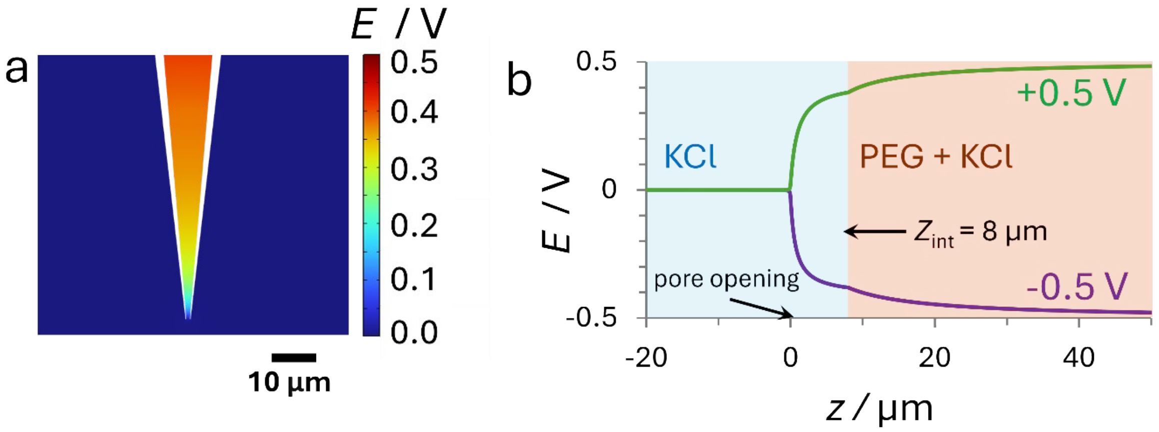

Figure S9. Simulated electric potential distribution. (a) Surface color plot of the electric potential distribution around the nanopipette tip region for E_{app} = 0.5 V, (b) Electric potential along the axis of symmetry (r = 0 nm) for +0.5 V (green curve) and -0.5 V (purple curve).

S5.3. Average ion concentrations along the symmetry axis under different applied potentials (PEG in nanopipette/KCl in bath)

Figure S10 shows the ion concentration distribution along the axis of the pipette as a function of the applied potential (-0.5 V to +0.5 V in steps of 0.1 V), which are qualitatively similar to those represented by the ±0.5 V curves represented in Figure 3 of the main text. Negative potentials show increased ionic concentrations within the pore with the maximum concentration at the PEG/KCl interface (Z_{int} = 8 μm) while positive potentials result in a depletion of the ion concentration with the minimum also at the interface. Increasing the magnitude of the applied voltage results in larger effects. The average ionic concentration is plotted due to the similar cation and anion concentration distributions (see discussion in 0).

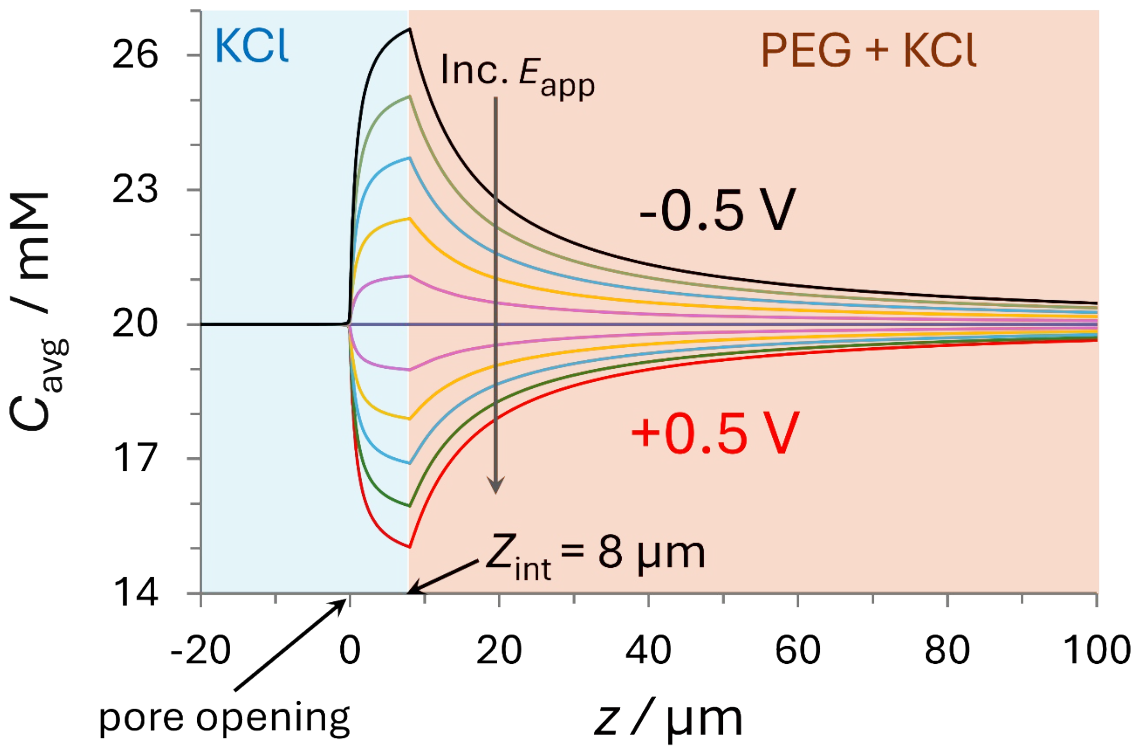

Figure S10. Average ion concentrations along the nanopipette axis of symmetry for PEG-in-Nanopipette / KCl-in-bath system using different applied potentials between –0.5 V and +0.5 V in steps of 0.1 V. The nanopore has a radius of 70 nm, and a half cone angle of 4.9°. Pipette fill solution: 25% w/v PEG 35K + 20 mM KCl; external bath solution: 20mM KCl.

S5.4. Average ion concentration profiles for KCl in nanopipette and external bath

Figure S10 shows the simulated ion concentration distribution at ±0.5 V applied potential biases for a pipette containing 20 mM of KCl immersed in a bath containing the same electrolyte. Similarly to Figure 2 of the main text, negative potentials show increased ionic concentrations within the pore while positive potentials result in a depletion of the ion concentration. Relative to Figure EGY, where the pipette fill solution is 25% w/v 35K PEG and the bath solution is 20 mM KCl, the extent of ion enrichment and depletion is low at -0.5 V and +0.5 V respectively. The potential dependent asymmetry in the ionic concentration distribution here is attributed to the surface charge and geometric asymmetry of the conical nanopore [10]. The average ionic concentration is plotted due to the similar cation and anion concentration distributions.

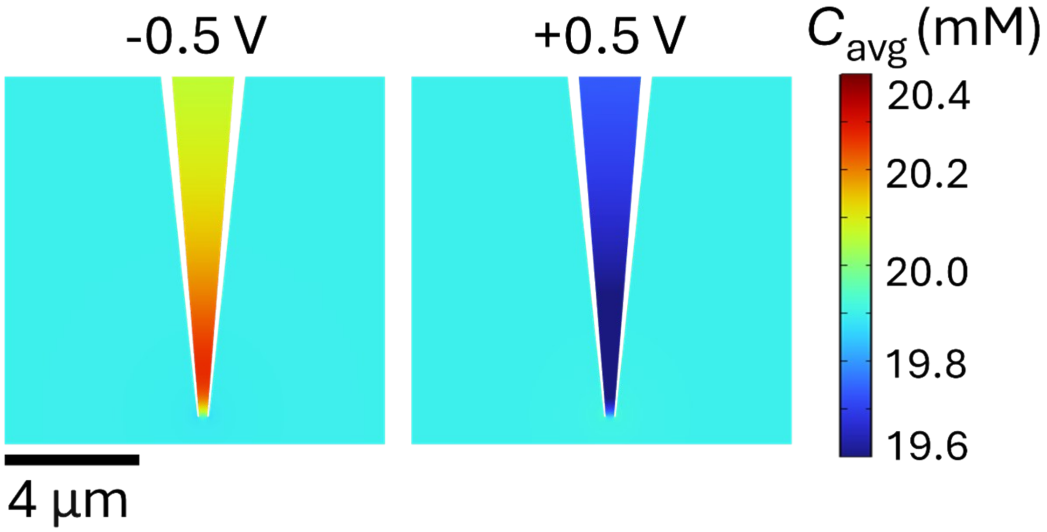

Figure S11. Plots of the simulated ion concentration ($C_{avg} = 1/2([K^+] + [Cl^-])$) close to the nanopipette tip at ±0.5 V for 20 mM KCl filled nanopipette in a bath filled with the same solution. Bulk concentration: 20 mM KCl. Nanopipette radius: 70 nm. Note that the concentration range is much smaller than the equivalent plots for 20 mM KCl + 25% w/v 35K PEG shown in Figure 3 of the main text.

S6. Detection of Ag nanoparticles with 70 nm radius nanopore

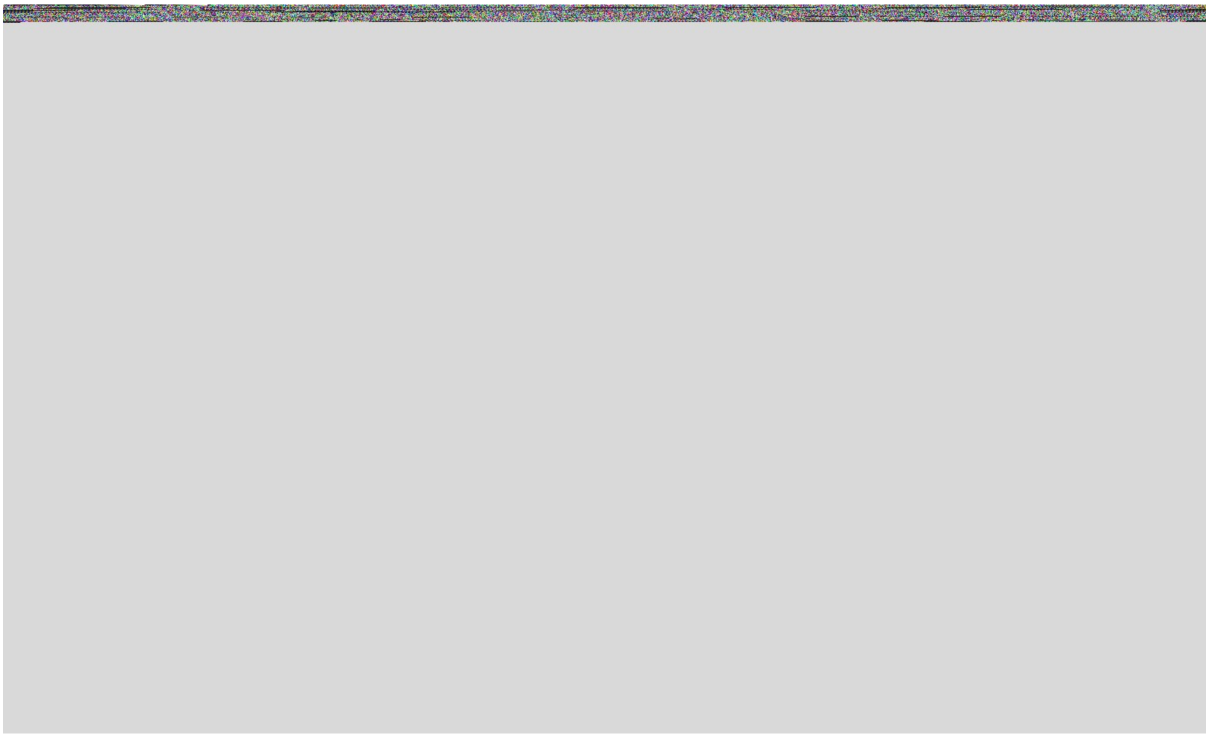

Figure S12. Scatter plot and ion current traces of Ag nanoparticles translocation using 70 nm radius nanopipette.

S7. Detection of Pt nanoparticles under different ionic strength

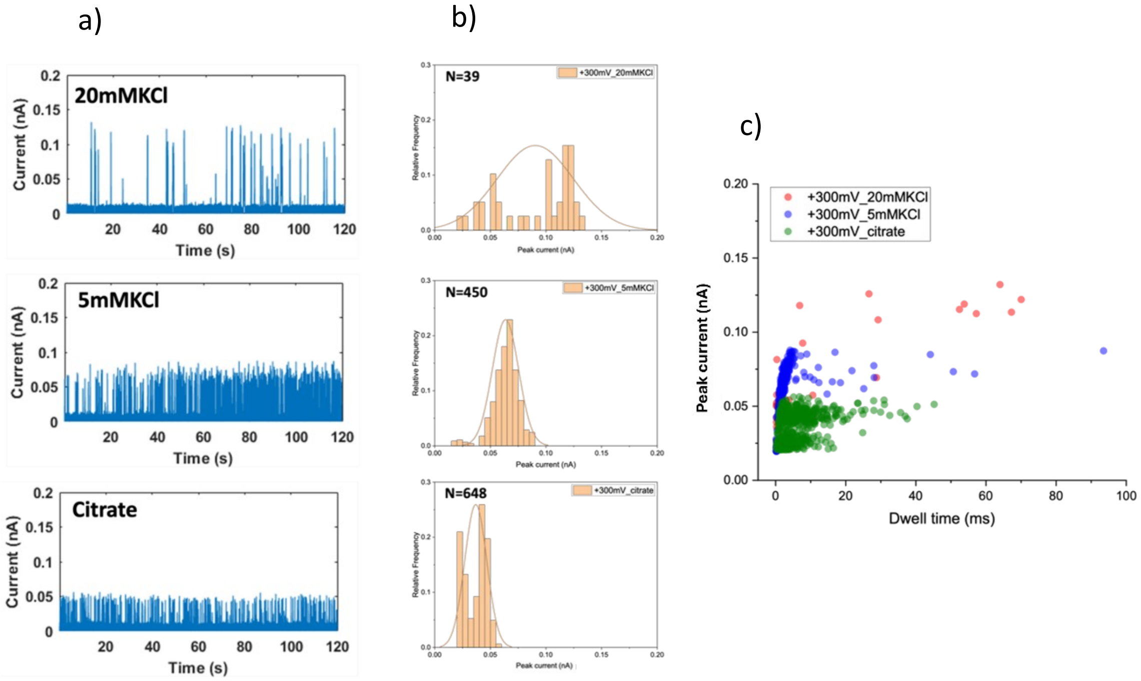

Figure S13. (a) Nanopore measurements of a solution of 30 nm citrate Pt NPs dissolved in either 20 mM KCL, 5 mM KCl, or in a sodium citrate solution using a 30nm glass nanopore with 25% PEG 35K and 20 mM KCl. c) Scatter plots of the single nanoparticle translocation events with nanoparticles dissolved in 20 mM KCl (red dots), 5 mM KCl (blue dots) and citrate (green dots). Reference electrode: Ag/AgCl frit filled with 20 mM KCl. Measurements performed with the Elements srl Nanopore reader at a sampling frequency of 20KHz.

S8. Confirmation of stability of nanoparticles in 20 mM KCl

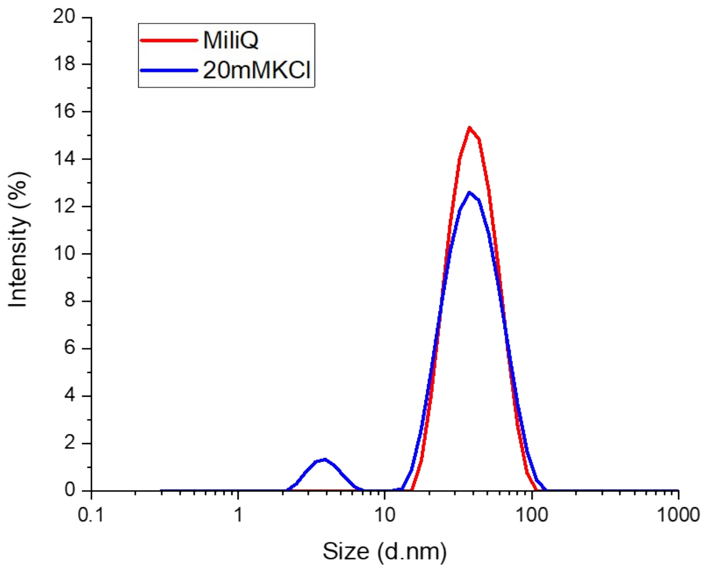

Figure S14. DLS spectra of the 30 nm citrate AgNPs in MilliQ water (red trace) and 20 mM KCl (blue trace)

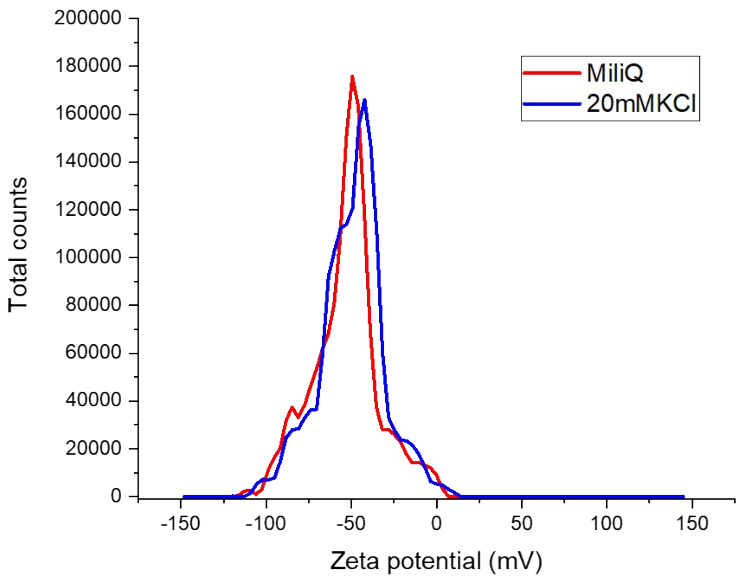

Figure S15. Z potential measurements of the 30 nm citrate AgNPs in MilliQ water(red trace) and 20 mM KCl (blue trace)

S9. References

1. F. Marcuccio, D. Soulias, C. C. C. Chau, S. E. Radford, E. Hewitt, P. Actis and M. A. Edwards, Mechanistic Study of the Conductance and Enhanced Single-Molecule Detection in a Polymer–Electrolyte Nanopore, *ACS Nanoscience Au*, 2023, **3**, 172-181.
2. H. S. W. Larry R. Faulkner, Allen J. Bard *Electrochemical Methods: Fundamentals and Applications*, John Wiley & Sons, 3rd edn., 2022.
3. C. Wei, A. J. Bard, G. Nagy and K. Toth, Scanning Electrochemical Microscopy. 28. Ion-Selective Neutral Carrier-Based Microelectrode Potentiometry, *Analytical Chemistry*, 2002, **67**, 1346-1356.
4. M. A. Edwards, C. G. Williams, A. L. Whitworth and P. R. Unwin, Scanning Ion Conductance Microscopy: a Model for Experimentally Realistic Conditions and Image Interpretation, *Analytical Chemistry*, 2009, **81**, 4482-4492.
5. N. Koizuim and T. Hanai, Dielectric Properties of Lower-membered Polyethylene Glycols at Low Frequencies, *The Journal of Physical Chemistry*, 2002, **60**, 1496-1500.
6. W. M. Haynes, ed., *CRC Handbook of Chemistry and Physics*, Boca Raton, 2016.
7. B. Zhang, Y. Zhang and H. S. White, Steady-State Voltammetric Response of the Nanopore Electrode, *Analytical Chemistry*, 2005, **78**, 477-483.
8. R. J. White, B. Zhang, S. Daniel, J. M. Tang, E. N. Ervin, P. S. Cremer and H. S. White, Ionic Conductivity of the Aqueous Layer Separating a Lipid Bilayer Membrane and a Glass Support, *Langmuir*, 2006, **22**, 10777-10783.

9. Handbook of Chemistry and Physics, https://hbcp.chemnetbase.com/contents/ContentsSearch.xhtml?dswid=2664, (accessed 05-07, 2024).

10. C. Wei, A. J. Bard and S. W. Feldberg, Current Rectification at Quartz Nanopipet Electrodes, *Analytical Chemistry*, 1997, **69**, 4627-4633.

11. W.-J. Lan, M. A. Edwards, L. Luo, R. T. Perera, X. Wu, C. R. Martin and H. S. White, Voltage-Rectified Current and Fluid Flow in Conical Nanopores, *Accounts of Chemical Research*, 2016, **49**, 2605-2613.

12. Z. Zhang, M. Ohl, S. O. Diallo, N. H. Jalarvo, K. Hong, Y. Han, G. S. Smith and C. Do, Dynamics of Water Associated with Lithium Ions Distributed in Polyethylene Oxide, *Physical Review Letters*, 2015, **115**, 198301.

13. C. Wen, S. Zeng, S. Li, Z. Zhang and S.-L. Zhang, On Rectification of Ionic Current in Nanopores, *Analytical Chemistry*, 2019, **91**, 14597-14604.
