# Supplementary material for: Multimodal nanoparticle analysis enabled by a polymer electrolyte nanopore combined with nanoimpact electrochemistry
Source: Faraday Discuss. 2024 Jul 22;257:303–15. doi: 10.1039/d4fd00143e (PMC11563348; doi:10.1039/d4fd00143e)
Supplement: FD-257-D4FD00143E-s002 [file FD-257-D4FD00143E-s002.pdf]

Supplementary Information (SI) for Faraday Discussions.
This journal is © The Royal Society of Chemistry 2024

Supporting Information for:

Multimodal nanoparticle analysis enabled by a polymer electrolyte nanopore combined with nanoimpact electrochemistry

Eugene Gyasi Agyemang,[a,b,#] Samuel Confederat,[c,d,#] Gayathri Mohanan,[c,d,#] Mahnaz Azimzadeh Sani,[e] Chalmers Chau,[c,d] Dylan Charnock,[c,d] Christoph Wälti,[c,d] Kristina Tschulik,[e] Martin Andrew Edwards[a,b*] and Paolo Actis[c,d*]

[a] Department of Chemistry and Biochemistry, University of Arkansas, Fayetteville, AR, 72701, USA

[b] Materials Science and Engineering, University of Arkansas, Fayetteville, AR, 72701, USA

[c] Bragg Centre for Materials Research, University of Leeds, LS2 9JT, UK

[d] School of Electronic and Electrical Engineering and Pollard Institute, University of Leeds, Leeds LS2 9JT, UK

[e] Ruhr University Bochum, Universitätsstraße 150, 44801 Bochum, Germany

# These authors contributed equally to the work

* Corresponding authors; maedw@uark.edu; p.actis@leeds.ac.uk

Below is a COMSOL-generated 'Model Report' representing the measurements where the bath contains 20 mM KCl (no PEG) and the nanopipette 20 mM KCl with 25% 35K-PEG. The PEG / KCl interface is set at 8 µm above the pore opening, with 25% PEG + 20mM KCl and 20mM KCl conditions in the nanopipette and external bath respectively. This model was used to create the data in Figures 2 and 3 in the main text and Figures S6, S7, S8, and S10 in the supporting information. Other variants of this model, which were generated by changing the conditions in the nanopipette and bath domains, were used to generate data for other nanopore measurements. The information presented below included all the boundary and subdomain conditions, mesh settings, solver settings, etc., necessary to recreate this model.

Glass nanopore

Report date	Jun 14, 2024, 3:41:57 PM

Author: Eugene Gyasi Agyemang

Contents

1 Global Definitions 4
1.1 Parameters 4
2 Model 1 8
2.1 Definitions 8
2.2 Nanopipette 21
2.3 Transport of Diluted Species 23
2.4 Electrostatics 33
2.5 Final Mesh 46
3 Steady State 52
3.1 Stationary 52
3.2 Solver Configurations 52
4 Results 54
4.1 Data Sets 54
4.2 Derived Values 55

1 Global Definitions

Date	Jun 14, 2024, 11:54:39 AM

GLOBAL SETTINGS

Name	ModelReportPEG,KCl.mph
Path	/scrfs/storage/egyasiag/home/ModelReportPEG,KCl.mph
Version	COMSOL Multiphysics 6.0 (Build: 354)

USED PRODUCTS

COMSOL Multiphysics
Chemical Reaction Engineering Module

COMPUTER INFORMATION

CPU	Intel(R) Xeon(R) Gold 6130 CPU @ 2.10GHz, 2 sockets
Operating system	Linux

1.1 PARAMETERS

PARAMETERS

Name	Expression	Value	Description
BathConductivity	3.86[mS/cm]	0.386 S/m	Experimentally measured conductivity of the bath medium
BeyondPoreRadius	PoreRadius*200*SizeFactor	1.42E−4 m	Radius for domain representing bath solution
PoreConductivity	1.19[mS/cm]	0.119 S/m	Experimentally measured conductivity of the pipette fill solution
cPore	20[mM]	20 mol/m^3	Concentration of the pipette fill solution
cbath	20[mM]	20 mol/m^3	Bath concentration
ConeApex	-PoreHeight*PoreRadius/(PoreTopRadius - PoreRadius)	−8.3062E−7 m	z value where the apex of the inner cone (if continued) would lie

Name	Expression	Value	Description
DClPore	0.55*PoreConductivity*(R_const*T)/(F_const^2*cbath)	8.568E−10 m²/s	Estimated diffusion coefficient of anions in pore (25% 35K PEG)
DClBath	0.51*BathConductivity*(R_const*T)/(F_const^2*cPore)	2.5771E−9 m²/s	CRC Handbook, 97th ed. Haynes, W. M., Ed CRC Press, 2016
DebyeLengthPore	sqrt((EpsilonRPore*epsilon0_const*R_const*T)/(2*cPore*F_const^2))	1.736E−9 m	Debye Length (pore)
DebyeLengthBath	sqrt((EpsilonRBath*epsilon0_const*R_const*T)/(2*cbath*F_const^2))	2.1532E−9 m	Debye Length (bath)
Delta	PoreRadius/50	1.42E−9 m	Used for selecting geometric boundaries
DKPore	0.45*PoreConductivity*(R_const*T)/(F_const^2*cbath)	7.0102E−10 m²/s	Estimated diffusion coefficient of anions in pore (25% PEG)
DKBath	0.49*BathConductivity*(R_const*T)/(F_const^2*cPore)	2.476E−9 m²/s	CRC Handbook, 97th ed. Haynes, W. M., Ed CRC Press, 2016
EpsilonRPore	52	52	Koizuim, N.; Hanai, T. Dielectric Properties of Lower-Membered Polyethylene Glycols at Low Frequencies. J. Phys. Chem. 1956
EpsilonRBath	80	80	CRC Handbook, 97th ed. Haynes, W. M., Ed CRC Press, 2016
gammaPore	(exp(1*e_const*GCSurfacePotentialPore/(2*k_B_const*T)) - 1)/(exp(1*e_const*GCSurfacePotential/(2*k_B_const*T)) + 1)	−0.074217	Helper for Gouy-Chapman (pore)

Name	Expression	Value	Description
gammaBath	(exp(1*e_const*GCSurfacePotentialBath/(2*k_B_const*T)) - 1)/(exp(1*e_const*GCSurfacePotentialBath/(2*k_B_const*T)) + 1)	−0.059951	Helper for Gouy-Chapman (bath)
GCSurfacePotentialPore	asinh(PoreSurfaceCharge/sqrt(8*cPore*N_A_const*EpsilonRPore*epsilon0_const*k_B_const*T))*(2*k_B_const*T)/(1*e_const)	−0.0075132 V	Surface potential difference from Gouy-Chapman (pore)
GCSurfacePotentialBath	asinh(PoreSurfaceCharge/sqrt(8*cbath*N_A_const*EpsilonRBath*epsilon0_const*k_B_const*T))*(2*k_B_const*T)/(1*e_const)	−0.0060651 V	Surface potential difference from Gouy-Chapman (bath)
PoreAngle	4.885666895[deg]	0.085271 rad	Inner truncated hollow cone half angle;
PoreHeight	50[um]*SizeFactor	5E−4 m	Truncated hollow cone's height in model; measured along the axis taking the aperture as flat
PoreRadius	71[nm]	7.1E−8 m	Inner cone aperture radius; measured from SEM imaging
PoreSurfaceCharge	-2[mC/m^2]	−0.002 C/m²	Surface charge of quartz glass wall, measured by best fit to experiment
PoreTopRadius	PoreRadius + tan(PoreAngle)*PoreHeight	4.281E−5 m	Estimated radius of the inner cone at z = PoreHeight
RAccessBottom	1/(4*BathConductivity*PoreRadius)	9.1221E6 Ω	Analytical expression for access resistance (assuming infinite RG and uniform concentration)
RadiusRatio	PoreRadius/PoreTopRadius	0.0016585	Ratio between the inner cone radii at the nanopipette

Name	Expression	Value	Description
			opening and at z = PoreHeight
RPore	1/(PoreConductivity*pi*PoreRadius*tan(PoreAngle))	4.4075E8 Ω	Analytical resistance inside the pore (assuming uniform conductivity PEG throughout)
RTot	RAccessBottom + RPore	4.4987E8 Ω	Total Resistance
SizeFactor	10	10	Scaling factor for selected boundaries dimensions
T	20[degC]	293.15 K	Temperature
VApp	0.5 [V]	0.5 V	Applied voltage
VBottom	VApp*RAccessBottom/RTot	0.010139 V	Potential drop outside the pore
WallAngle	atan((WallTopRadius - WallRadius)/PoreHeight)	0.11507 rad	Estimated half cone angle of the outer cone (glass wall)
WallRadius	PoreRadius + WallThickness	9.6E−8 m	Outer cone (glass wall) radius at z = 0
WallThickness	25[nm]	2.5E−8 m	Glass thickness at the nanopipette opening; from SEM
WallTopRadius	WallRadius/RadiusRatio	5.7884E−5 m	Outer cone (glass wall) radius at z = PoreHeight
Z_int	8 [um]	8E−6 m	Interface vertical distance within the nanopipette

2 Model 1

SETTINGS

Description	Value
Unit system	Same as global system (SI)
Avoid inverted elements by curving interior domain elements	Off

2.1 DEFINITIONS

2.1.1 Variables

Excess Charge

SELECTION

Geometric entity level	Domain
Selection	Geometry geom1: Dimension 2: Domains 1–4

Name	Expression	Unit	Description
ExcessCharge	F_const*(cK - cCl)	C/m³	

Distance from glass (internal electrode)

SELECTION

Geometric entity level	Boundary
Name	Internal Electrode
Selection	Named sel3: Geometry geom1: Dimension 1: Boundary 6

Name	Expression	Unit	Description
dGlass	(PoreTopRadius - r)*sin(90[deg] - PoreAngle)	m	

Distance from glass (external electrode, upper part)

SELECTION

Geometric entity level	Boundary
Selection	Geometry geom1: Dimension 1: Boundaries 13–14

Name	Expression	Unit	Description
dGlass	cos(WallAngle)*(r - WallRadius - z*tan(WallAngle))	m	

Distance from glass (external electrode, bottom part)

SELECTION

Geometric entity level	Boundary
Selection	Geometry geom1: Dimension 1: Boundary 12

Name	Expression	Unit	Description
dGlass	(r< PoreRadius)*(sqrt((r - PoreRadius)^2 + z^2)) + (-z)*(r>=PoreRadius)*(r<WallRadius) + (r>=WallRadius)*(sqrt((r - WallRadius)^2 + z^2))	m	

Analytical Potential, No Charge (Bath)

SELECTION

Geometric entity level	Domain
Selection	Geometry geom1: Dimension 2: Domains 1, 3–4

Name	Expression	Unit	Description
AnalyticalPotentialNoCharge	DiskElec(r, -z, PoreRadius)*VBottom	V	

Analytical Potential, No Charge (Pore)

SELECTION

Geometric entity level	Domain
Selection	Geometry geom1: Dimension 2: Domain 2

Name	Expression	Unit	Description
AnalyticalPotentialNoCharge	VBottom + (VApp - VBottom)*1/(PoreConductivity*pi)*z/(PoreRadius*(PoreRadius + z/PoreHeight*(PoreTopRadius - PoreRadius)))/RPore	V	

Domain Expressions Gouy-Chapman

SELECTION

Geometric entity level	Entire model

Name	Expression	Unit	Description
cCl_GC	c_GC(WallDistance, -1)	mol/m^3	
cK_GC	c_GC(WallDistance, 1)	mol/m^3	
Potential_GC	E_GC(WallDistance)	V	
cCl_GCBath	c_GCBath(WallDistance, -1)	mol/m^3	
cK_GCBath	c_GCBath(WallDistance, 1)	mol/m^3	
Potential_GCBath	E_GCBath(WallDistance)	V	

Wall Distance (Pore)

SELECTION

Geometric entity level	Domain
Selection	Geometry geom1: Dimension 2: Domains 2–3

Name	Expression	Unit	Description
lambda	(r*PoreRadius - z*ConeApex + ConeApex^2)/(PoreRadius^2 + ConeApex^2)		helper value for point of nearest wall
WallDistance	(lambda>=1)*sqrt((r - lambda*PoreRadius)^2 + (z - (ConeApex - lambda*ConeApex))^2) + (lambda<1)*sqrt((r - PoreRadius)^2 + z^2)	m	

Wall Distance (Bath, bottom part)

SELECTION

Geometric entity level	Domain
Selection	Geometry geom1: Dimension 2: Domain 1

Name	Expression	Unit	Description
WallDistance	(r< PoreRadius)*(sqrt((r - PoreRadius)^2 + z^2)) + (-z)*(r>=PoreRadius)*(r<WallRadius) + (r>=WallRadius)*(sqrt((r - WallRadius)^2 + z^2))	m	

Wall Distance (Bath, upper part)

SELECTION

Geometric entity level	Domain
Selection	Geometry geom1: Dimension 2: Domain 4

Name	Expression	Unit	Description
WallDistance	cos(WallAngle)*(r - WallRadius - z*tan(WallAngle))	m	

2.1.2 Functions

Gouy-Chapman Potential (Pore)

Function name	E_GC
Function type	Analytic

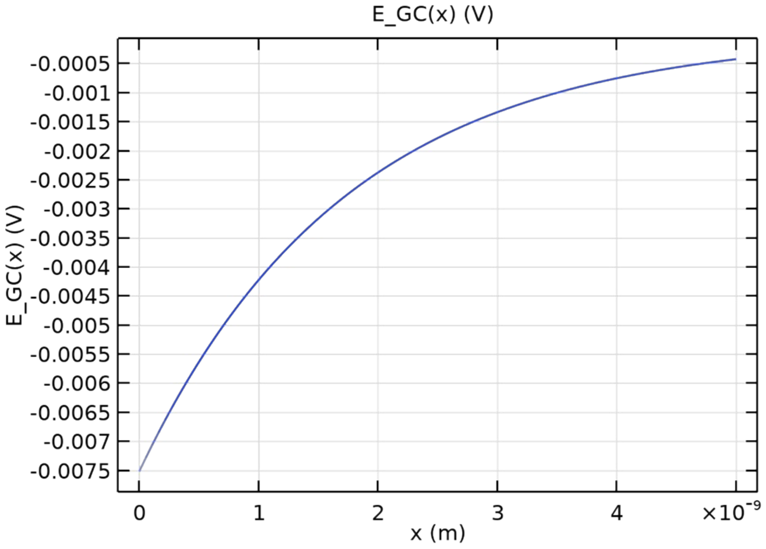

Gouy-Chapman Potential (Pore)

DEFINITION

Description	Value
Expression	(2*k_B_const*T/e_const)*log((1 + gammaPore*exp(-x/DebyeLengthPore))/ (1 - gammaPore*exp(-x/DebyeLengthPore)))
Arguments	x

UNITS

Description	Value
Function	V

UNITS

Argument	Unit
x	m

Gouy-Chapman Potential (Bath)

Function name	E_GCBath
Function type	Analytic

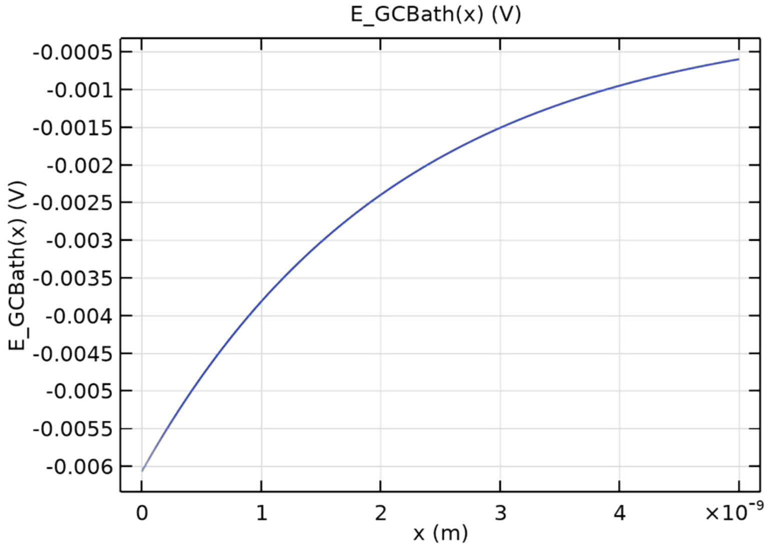

Gouy-Chapman Potential (Bath)

DEFINITION

Description	Value
Expression	((2*k_B_const*T/e_const)*log((1 + gammaBath*exp(-x/DebyeLengthBath))/ (1 - gammaBath*exp(-x/DebyeLengthBath))))
Arguments	x

UNITS

Description	Value
Function	V

UNITS

Argument	Unit
x	m

Gouy-Chapman Concentration (Pore)

Function name	c_GC
Function type	Analytic

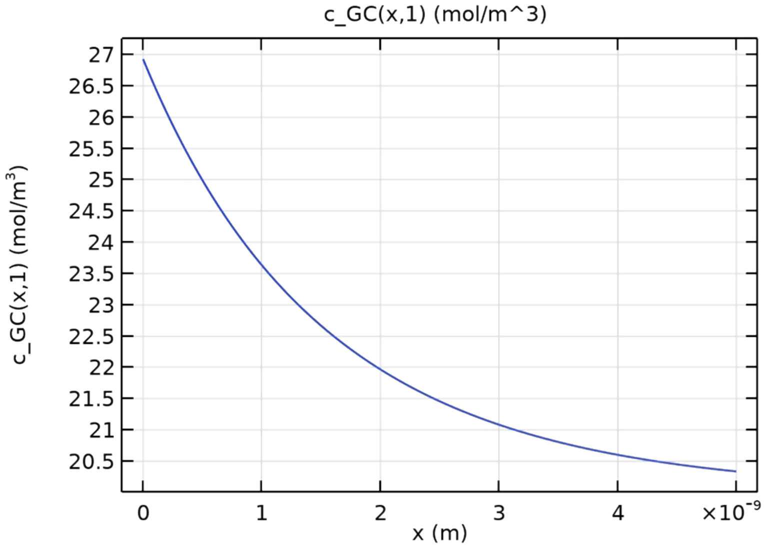

Gouy-Chapman Concentration (Pore)

DEFINITION

Description	Value
Expression	cPore*exp(-z*e_const*E_GC(x)/(k_B_const*T))
Arguments	{x, z}

UNITS

Description	Value
Function	mol/m^3

UNITS

Argument	Unit
x	m
z	1

Gouy-Chapman Concentration (Bath)

Function name	c_GCBath
Function type	Analytic

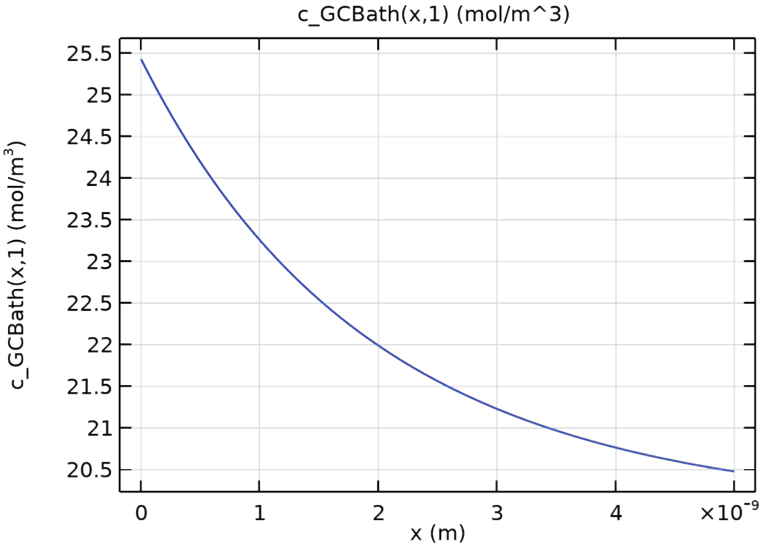

Gouy-Chapman Concentration (Bath)

DEFINITION

Description	Value
Expression	cbath*exp(-z*e_const*E_GCBath(x)/(k_B_const*T))
Arguments	{x, z}

UNITS

Description	Value
Function	mol/m^3

UNITS

Argument	Unit
x	m
z	1

Disk Electrode (Normalized Distribution)

Function name	DiskElec
Function type	Analytic

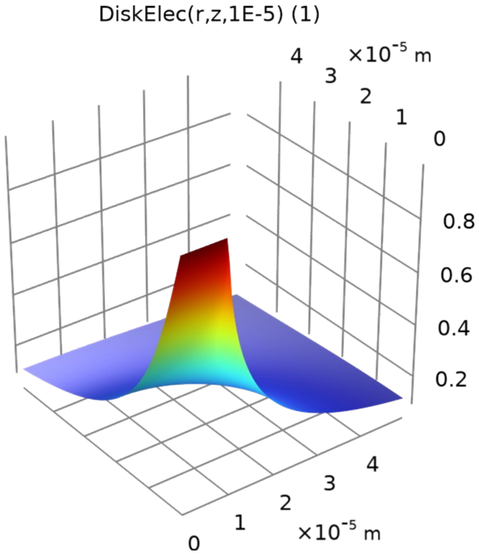

Disk Electrode (Normalized Distribution)

DEFINITION

Description	Value
Expression	2/pi*asin(2*a/(sqrt((z + eps)^2 + (a + eps + r)^2) + sqrt((z + eps)^2 + (a + eps - r)^2)))
Arguments	{r, z, a}

UNITS

Description	Value
Function	1

UNITS

Argument	Unit
r	m
z	m
a	m

2.1.3 Selections

2.1.3.1 Symmetry Axis

Selection type
Box

Selection
Boundaries 1–2, 4

GEOMETRIC ENTITY LEVEL

Description	Value
Level	Boundary

OUTPUT ENTITIES

Description	Value
Include entity if	All vertices inside box

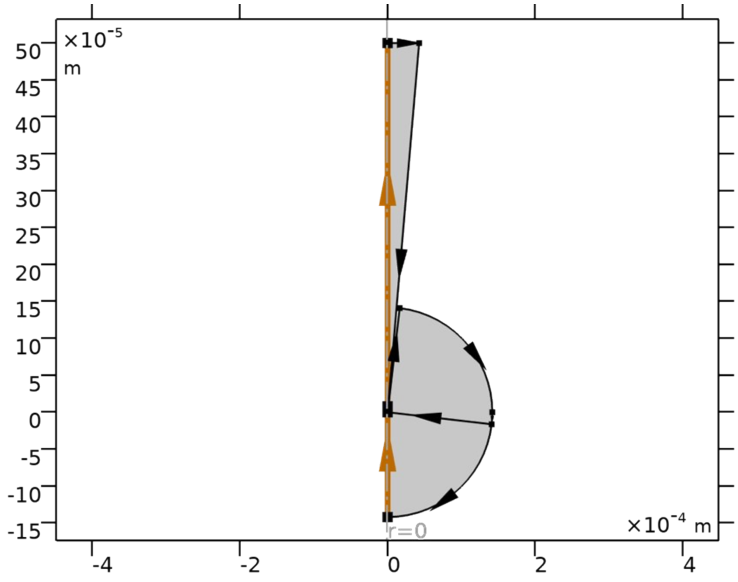

Symmetry Axis

2.1.3.2 Glass Wall

Selection type
Explicit

Selection
Boundaries 7–9, 11

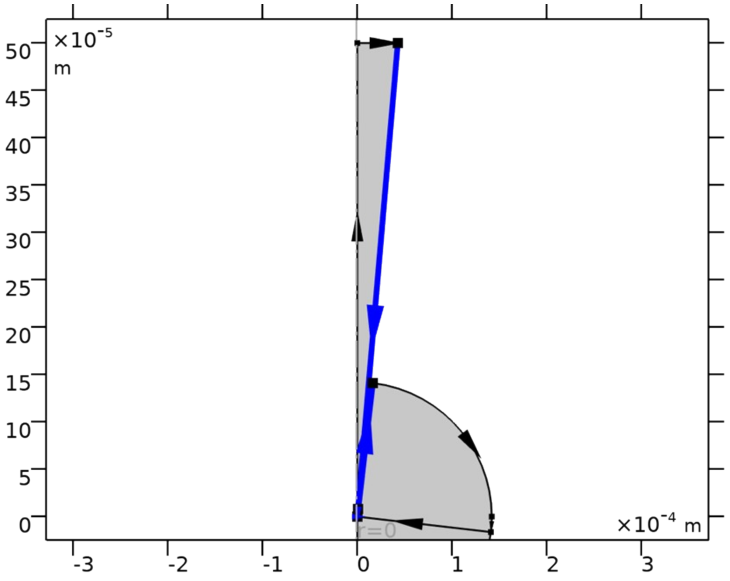

Glass Wall

2.1.3.3 Aperture point

Selection type
Explicit

Selection
Point 5

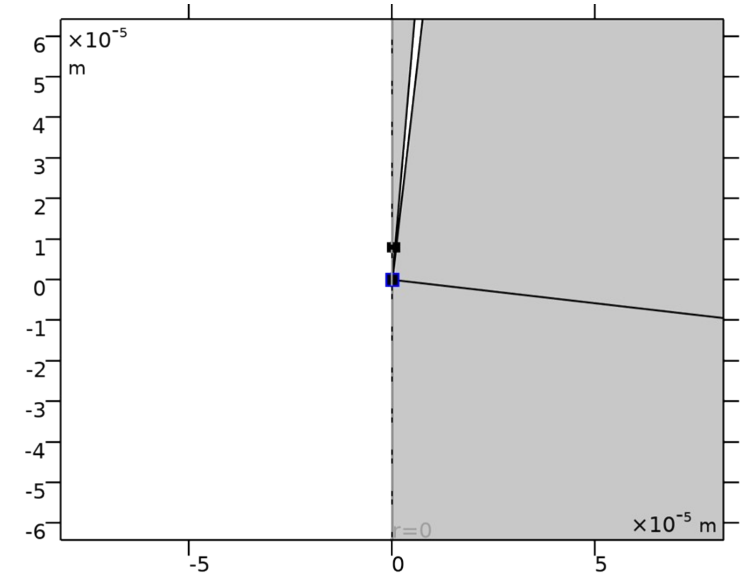

Aperture point

2.1.3.4 Internal Electrode

Selection type
Explicit

Selection
Boundary 6

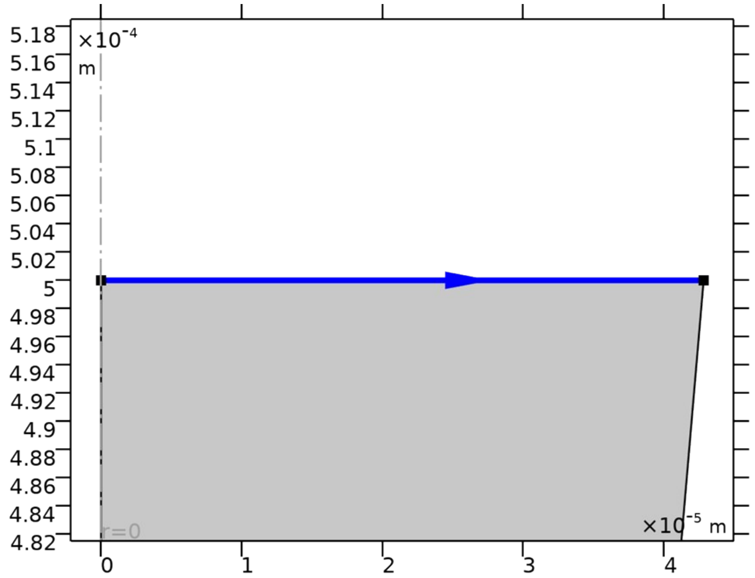

Internal Electrode

2.1.3.5 External Electrode

Selection type
Explicit

Selection
Boundaries 12–14

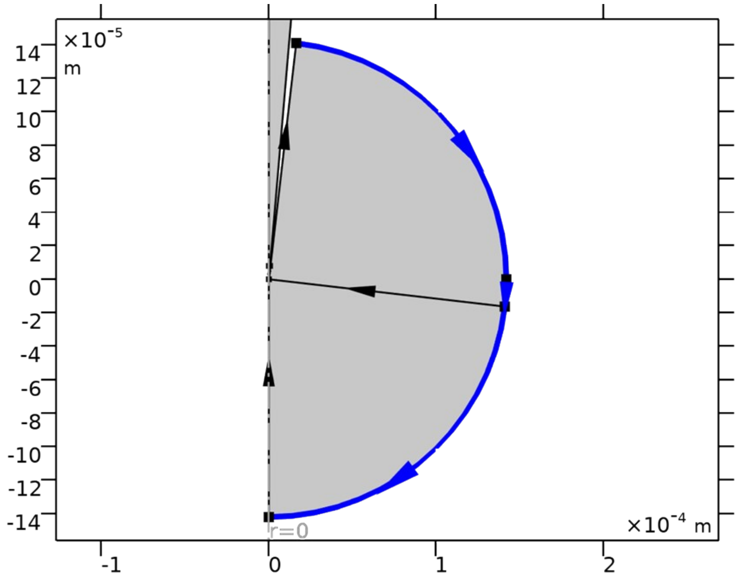

External Electrode

2.1.4 Probes

Current (internal electrode)

Probe type	Boundary probe

SELECTION

Geometric entity level	Boundary
Selection	Geometry geom1: Dimension 1: Boundary 6

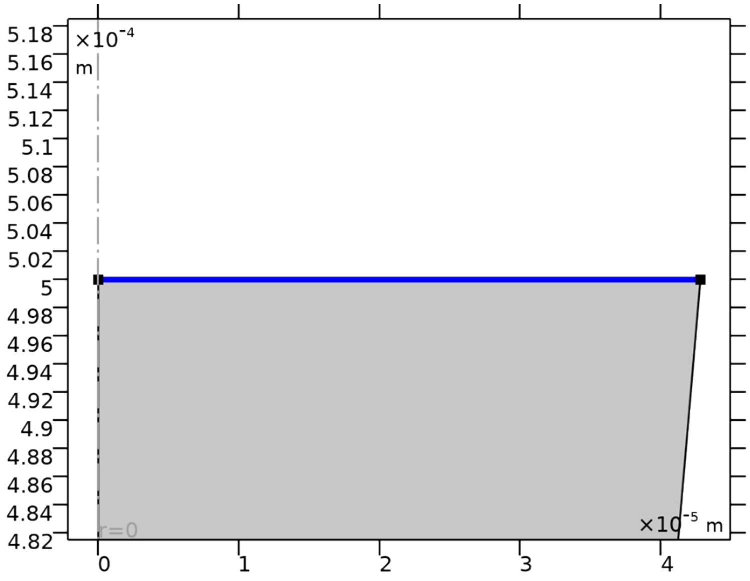

Selection

PROBE TYPE

Description	Value
Type	Integral

EXPRESSION

Description	Value
Expression	-F_const*(chds.bndFlux_cK - chds.bndFlux_cCl)*2*pi*r
Table and plot unit	nA
Description	Current (internal)

TABLE AND WINDOW SETTINGS

Description	Value
Output table	Probe Table 1
Plot window	Probe Plot 3

2.1.5 Coordinate Systems

Boundary System 1

Coordinate system type	Boundary system
Tag	sys1

COORDINATE NAMES

First	Second	Third
t1	to	n

2.2 NANOPIPETTE

Pore with glass of infinite width

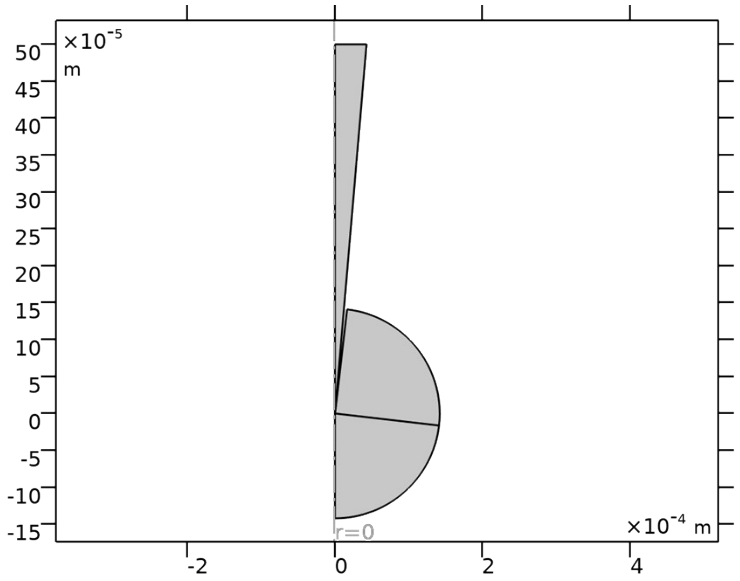

Nanopipette

UNITS

Length unit	m
Angular unit	deg

GEOMETRY STATISTICS

Description	Value
Space dimension	2
Number of domains	4
Number of boundaries	14
Number of vertices	11

2.2.1 Pore (b5)

POLYGON SEGMENTS

Description	Value
Control points	{{0, 0, PoreTopRadius, PoreRadius, 0}, {0, PoreHeight, PoreHeight, 0, 0}}
Degree	{1, 1, 1, 1}
Weights	{1, 1, 1, 1, 1, 1, 1, 1}
Type	Solid

2.2.2 Wall (pol1)

OBJECT TYPE

Description	Value
Type	Open curve

COORDINATES

Description	Value
Data source	Table

COORDINATES

r (m)	z (m)
PoreRadius	0
WallRadius	0
WallTopRadius	PoreHeight

2.2.3 Bath (c1)

SELECTIONS OF RESULTING ENTITIES

Description	Value
Resulting objects selection	On

POSITION

Description	Value
Position	{0, 0}

ROTATION ANGLE

Description	Value
Rotation	-90

SIZE AND SHAPE

Description	Value
Radius	BeyondPoreRadius
Sector angle	180

2.2.4 Difference Bath from Pipette & Wall (dif1)

SETTINGS

Description	Value
Keep objects to add	On
Keep objects to subtract	On

2.2.5 Rectangle for dGlass (r1)

POSITION

Description	Value
Position	{WallRadius, 0}

ROTATION ANGLE

Description	Value
Rotation	-WallAngle

SIZE

Description	Value
Width	BeyondPoreRadius
Height	BeyondPoreRadius

2.2.6 Z_int Point 1 (pt1)

POINT

Description	Value
Point coordinate	{0, 8.0E-6}

2.2.7 Z_int Point 2 (pt2)

POINT

Description	Value
Point coordinate	{7.548259896776561E-7, 8.0E-6}

2.3 TRANSPORT OF DILUTED SPECIES

USED PRODUCTS

COMSOL Multiphysics
Chemical Reaction Engineering Module

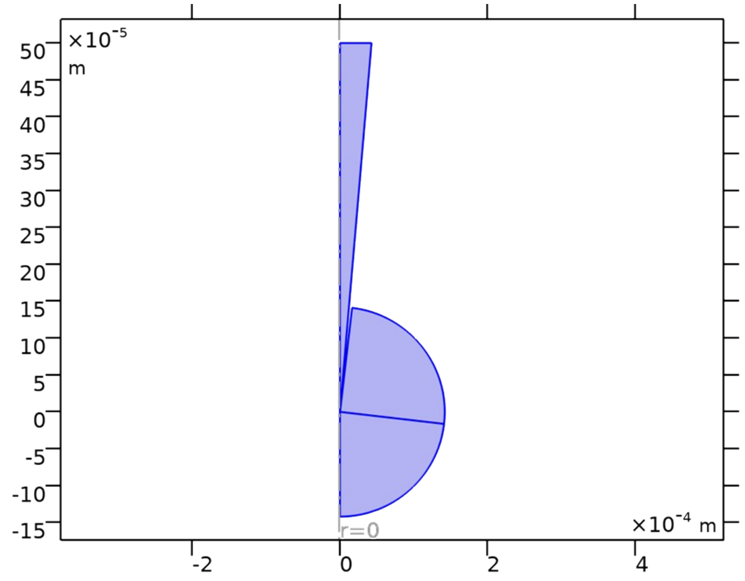

Transport of Diluted Species

SELECTION

Geometric entity level	Domain
Selection	Geometry geom1: Dimension 2: All domains

EQUATIONS

$$\nabla \cdot \mathbf{J}_i = R_i$$
$$\mathbf{J}_i = -D_i \nabla c_i - z_i u_{m,i} F c_i \nabla V$$

2.3.1 Interface Settings

Discretization

SETTINGS

Description	Value
Concentration	Quadratic

SETTINGS

Description	Value
Equation form	Study controlled

Advanced Settings

SETTINGS

Description	Value
Convective term	Conservative form

Transport Mechanisms

SETTINGS

Description	Value
Convection	Off
Migration in electric field	On
Mass transfer in porous media	Off

2.3.2 Diffusion and Migration (Pore)

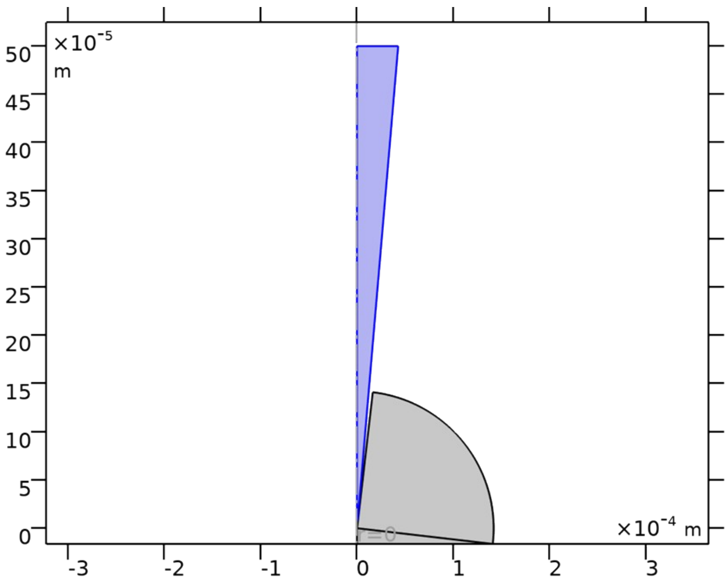

Diffusion and Migration (Pore)

SELECTION

Geometric entity level	Domain
Selection	Geometry geom1: Dimension 2: All domains

EQUATIONS

$$\nabla \cdot \mathbf{J}_i = R_i$$

$$\mathbf{J}_i = -D_i \nabla c_i - z_i u_{m,i} F c_i \nabla V$$

Diffusion

SETTINGS

Description	Value
Source	Material
Material	None
Diffusion coefficient	User defined

Description	Value
Diffusion coefficient	{{DKPore, 0, 0}, {0, DKPore, 0}, {0, 0, DKPore}}
Diffusion coefficient	User defined
Diffusion coefficient	{{DClPore, 0, 0}, {0, DClPore, 0}, {0, 0, DClPore}}

Migration in Electric Field

SETTINGS

Description	Value
Electric potential	Electric potential (es)
Mobility	Nernst - Einstein relation
Charge number	{1, -1}

Coordinate System Selection

SETTINGS

Description	Value
Coordinate system	Global coordinate system

Model Input

SETTINGS

Description	Value
Temperature	User defined
Temperature	T

2.3.3 Axial Symmetry

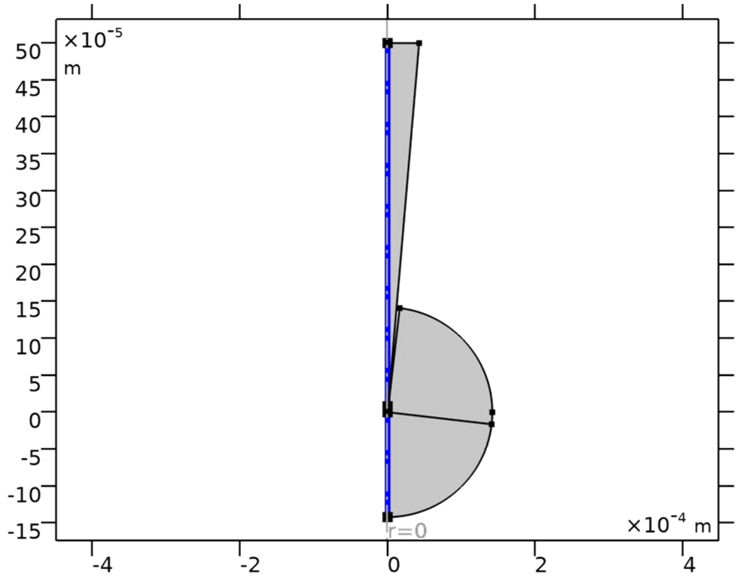

Axial Symmetry

SELECTION

Geometric entity level	Boundary
Selection	Geometry geom1: Dimension 1: All boundaries

2.3.4 No Flux (Glass Wall)

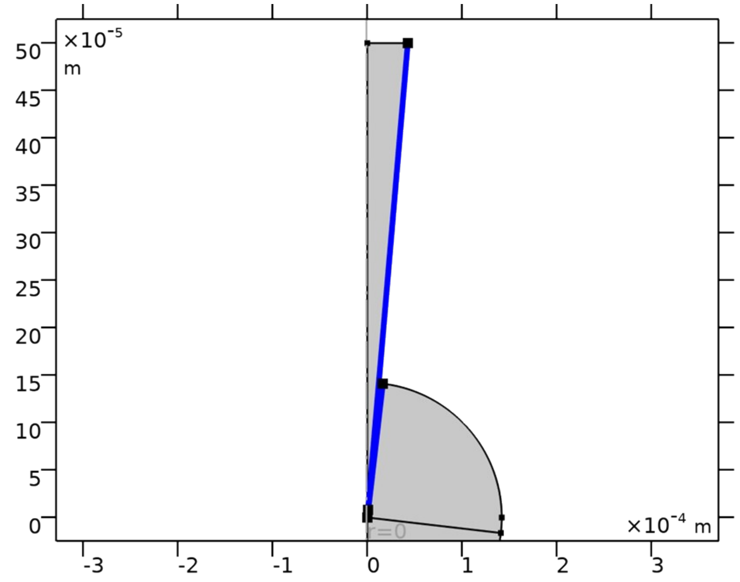

No Flux (Glass Wall)

SELECTION

Geometric entity level	Boundary
Selection	Geometry geom1: Dimension 1: All boundaries

EQUATIONS

$$-\mathbf{n} \cdot \mathbf{J}_i = 0$$

2.3.5 Initial Values

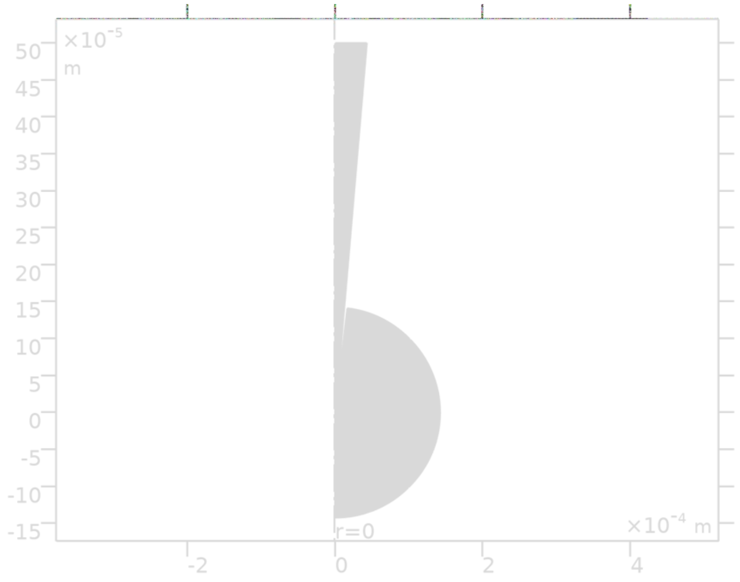

Initial Values

SELECTION

Geometric entity level	Domain
Selection	Geometry geom1: Dimension 2: All domains

Initial Values

SETTINGS

**Description**	**Value**
Concentration	{cPore, cPore}

2.3.6 Diffusion and Migration (Bath)

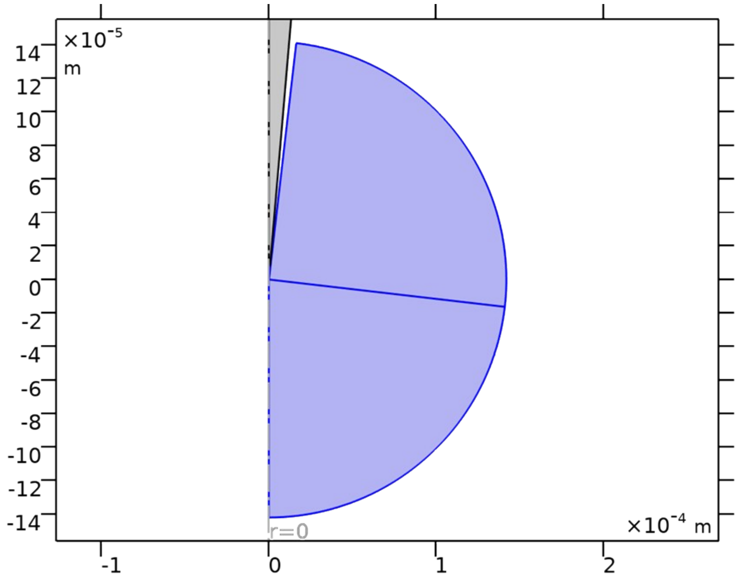

Diffusion and Migration (Bath)

SELECTION

Geometric entity level	Domain
Selection	Geometry geom1: Dimension 2: Domains 1–2, 4

EQUATIONS

$$\nabla \cdot \mathbf{J}_i = R_i$$
$$\mathbf{J}_i = -D_i \nabla c_i - z_i u_{m,i} F c_i \nabla V$$

Diffusion

SETTINGS

**Description**	**Value**
Source	Material
Material	None
Diffusion coefficient	User defined
Diffusion coefficient	{{DKBath, 0, 0}, {0, DKBath, 0}, {0, 0, DKBath}}
Diffusion coefficient	User defined
Diffusion coefficient	{{DClBath, 0, 0}, {0, DClBath, 0}, {0, 0, DClBath}}

Migration in Electric Field

SETTINGS

**Description**	**Value**
Electric potential	Electric potential (es)

Description	Value
Mobility	Nernst - Einstein relation
Charge number	{1, -1}

Coordinate System Selection

SETTINGS

Description	Value
Coordinate system	Global coordinate system

Model Input

SETTINGS

Description	Value
Temperature	User defined
Temperature	T

2.3.7 Initial Values (Gouy-Chapman, Bath)

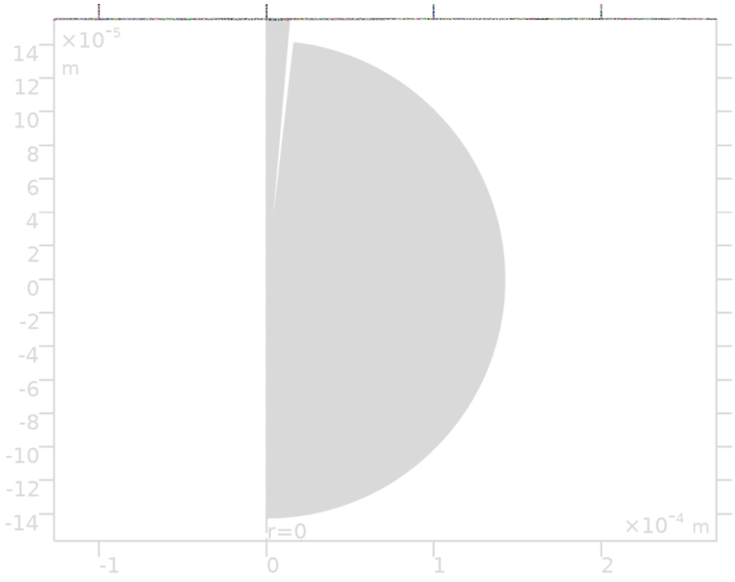

Initial Values (Gouy-Chapman, Bath)

SELECTION

Geometric entity level	Domain
Selection	Geometry geom1: Dimension 2: Domains 1–2, 4

Initial Values

SETTINGS

Description	Value

Description	Value
Concentration	{cK_GCBath, cCl_GCBath}

2.3.8 Initial Values (Gouy-Chapman, Pore)

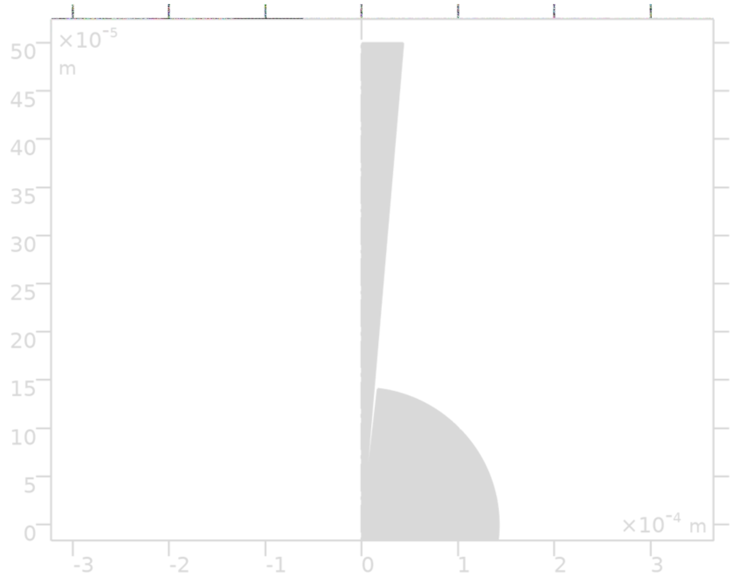

Initial Values (Gouy-Chapman, Pore)

SELECTION

Geometric entity level	Domain
Selection	Geometry geom1: Dimension 2: Domain 3

Initial Values

SETTINGS

Description	Value
Concentration	{cK_GC, cCl_GC}

2.3.9 Concentration (Gouy-Chapman, internal electrode)

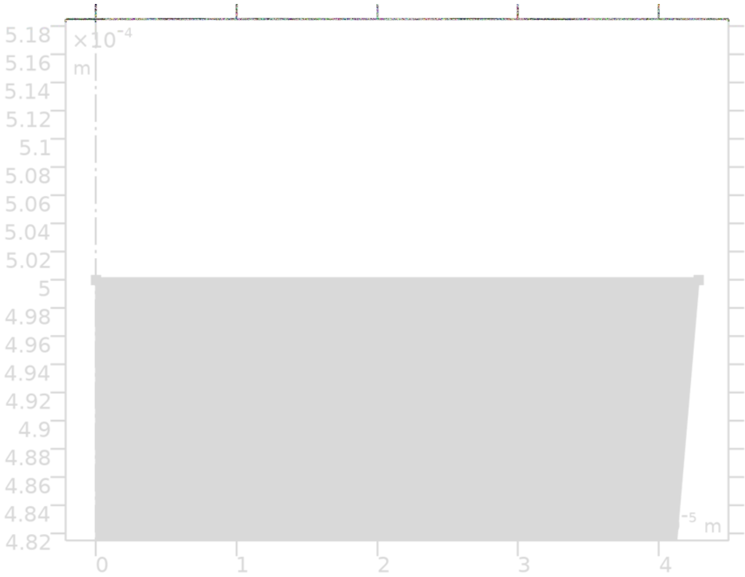

Concentration (Gouy-Chapman, internal electrode)

SELECTION

Geometric entity level	Boundary
Selection	Geometry geom1: Dimension 1: Boundary 6

EQUATIONS

$$c_i = c_{0,j}$$

Concentration

SETTINGS

Description	Value
Species cK	On
Species cCl	On
Concentration	{c_GC(dGlass, 1), c_GC(dGlass, -1)}

2.3.10 Concentration (Gouy-Chapman, external electrode)

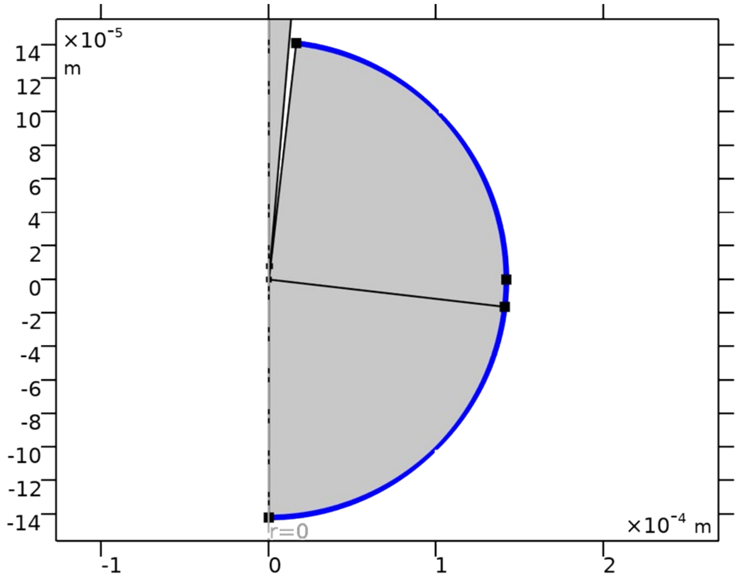

Concentration (Gouy-Chapman, external electrode)

SELECTION

Geometric entity level	Boundary
Selection	Geometry geom1: Dimension 1: Boundaries 12–14

EQUATIONS

$$c_i = c_{0,i}$$

Concentration

SETTINGS

**Description**	**Value**
Species cK	On
Species cCl	On
Concentration	{c_GCBath(dGlass, 1), c_GCBath(dGlass, -1)}

2.4 ELECTROSTATICS

USED PRODUCTS

COMSOL Multiphysics

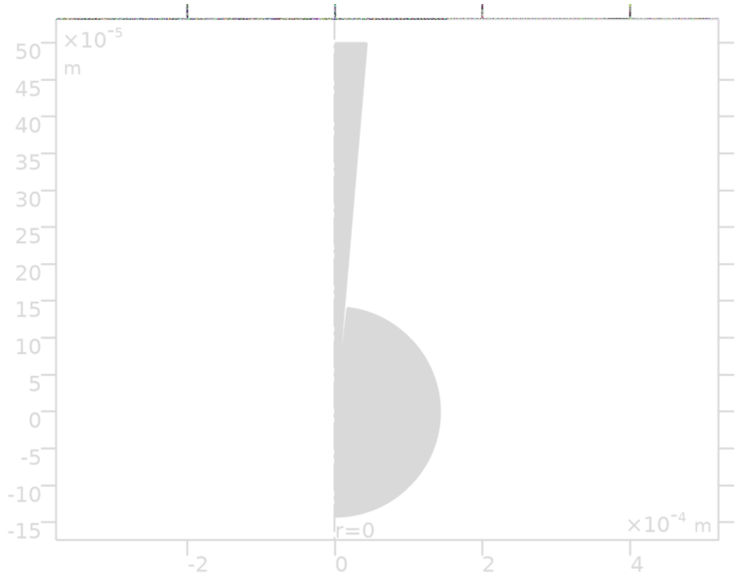

Electrostatics

SELECTION

Geometric entity level	Domain
Selection	Geometry geom1: Dimension 2: All domains

EQUATIONS

$$\nabla \cdot \mathbf{D} = \rho_{\mathrm{v}}$$

$$\mathbf{E} = -\nabla V$$

2.4.1 Interface Settings

Discretization

SETTINGS

**Description**	**Value**
Electric potential	Quadratic

Manual Terminal Sweep Settings

SETTINGS

**Description**	**Value**
Use manual terminal sweep	Off
Reference impedance	50[ohm]

2.4.2 Charge Conservation (Pore)

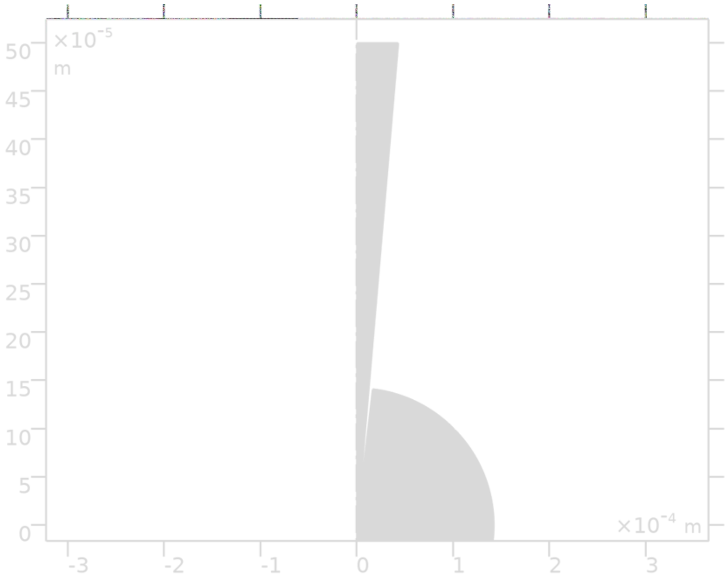

Charge Conservation (Pore)

SELECTION

Geometric entity level	Domain
Selection	Geometry geom1: Dimension 2: All domains

EQUATIONS

$$\mathbf{E} = -\nabla V$$

$$\nabla \cdot (\epsilon_0 \epsilon_r \mathbf{E}) = \rho_v$$

Constitutive Relation D-E

SETTINGS

Description	Value
Dielectric model	Relative permittivity
Relative permittivity	User defined
Relative permittivity	{{EpsilonRPore, 0, 0}, {0, EpsilonRPore, 0}, {0, 0, EpsilonRPore}}

Coordinate System Selection

SETTINGS

Description	Value
Coordinate system	Global coordinate system

Axial Symmetry

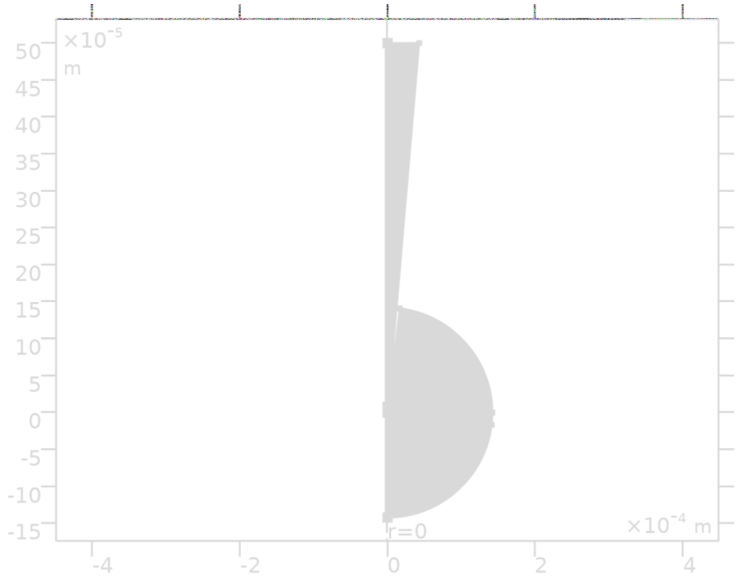

Axial Symmetry

SELECTION

Geometric entity level	Boundary
Selection	Geometry geom1: Dimension 1: All boundaries

2.4.3 Zero Charge

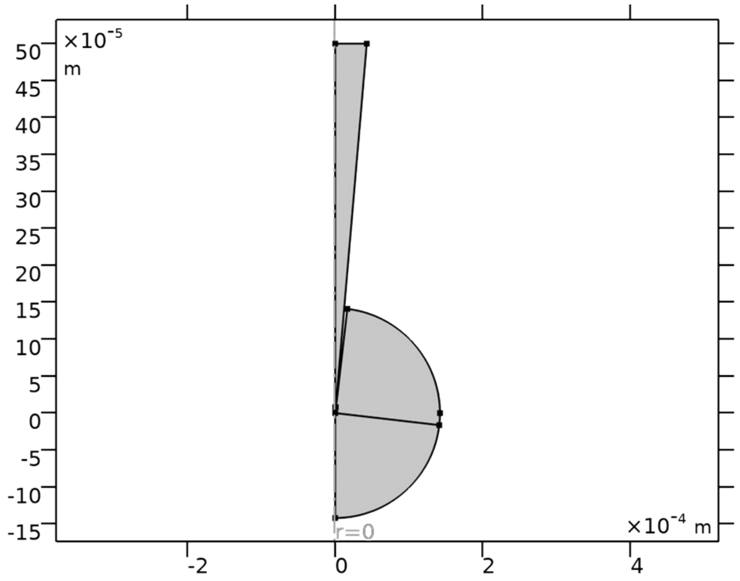

Zero Charge

SELECTION

Geometric entity level	Boundary

Selection	Geometry geom1: Dimension 1: All boundaries

EQUATIONS

$$\mathbf{n} \cdot \mathbf{D} = 0$$

2.4.4 Initial Values

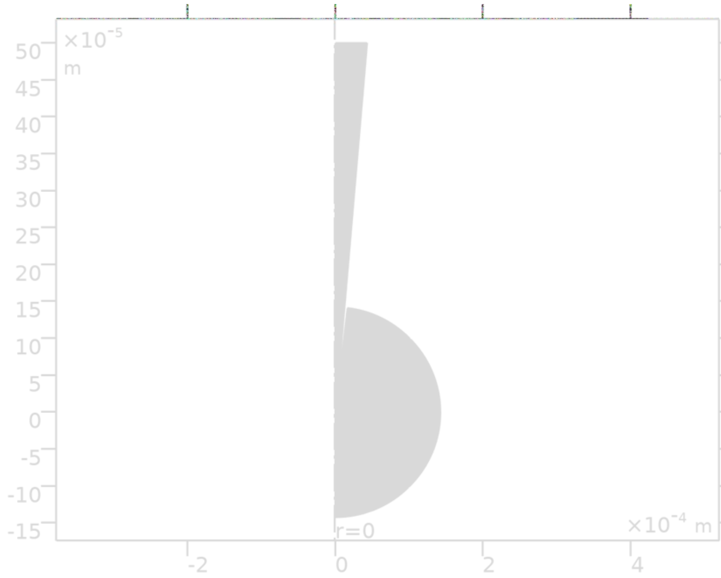

Initial Values

SELECTION

Geometric entity level	Domain
Selection	Geometry geom1: Dimension 2: All domains

SETTINGS

Description	Value
Electric potential	0

2.4.5 Charge Conservation (Bath)

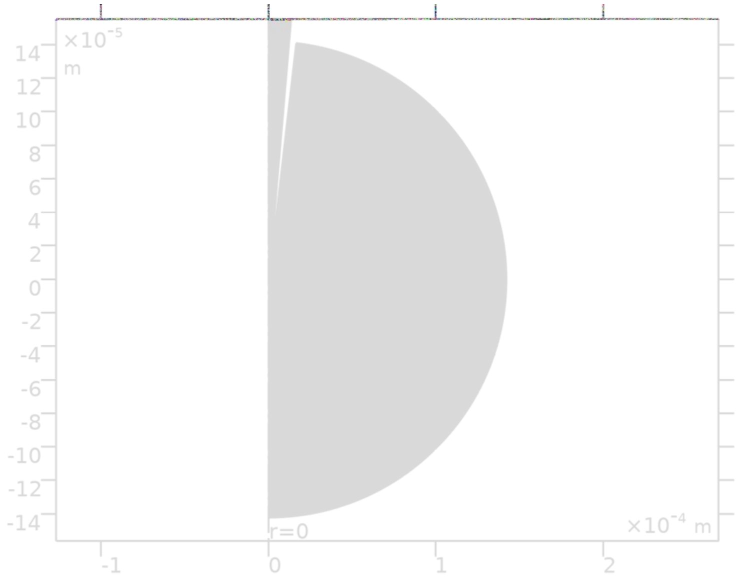

Charge Conservation (Bath)

SELECTION

Geometric entity level	Domain
Selection	Geometry geom1: Dimension 2: Domains 1–2, 4

EQUATIONS

$$\mathbf{E} = -\nabla V$$
$$\nabla \cdot (\epsilon_0 \epsilon_r \mathbf{E}) = \rho_v$$

Constitutive Relation D-E

SETTINGS

Description	Value
Dielectric model	Relative permittivity
Relative permittivity	User defined
Relative permittivity	{{EpsilonRBath, 0, 0}, {0, EpsilonRBath, 0}, {0, 0, EpsilonRBath}}

Coordinate System Selection

SETTINGS

Description	Value
Coordinate system	Global coordinate system

2.4.6 Initial Values (Gouy-Chapman in Pore)

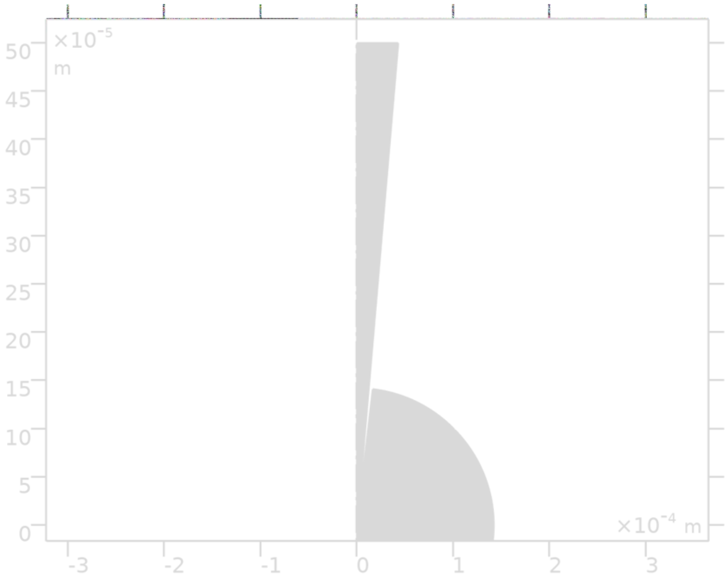

Initial Values (Gouy-Chapman in Pore)

SELECTION

Geometric entity level	Domain
Selection	Geometry geom1: Dimension 2: Domain 3

SETTINGS

Description	Value
Electric potential	Potential_GC + AnalyticalPotentialNoCharge

2.4.7 Initial Values (Gouy-Chapman in Bath)

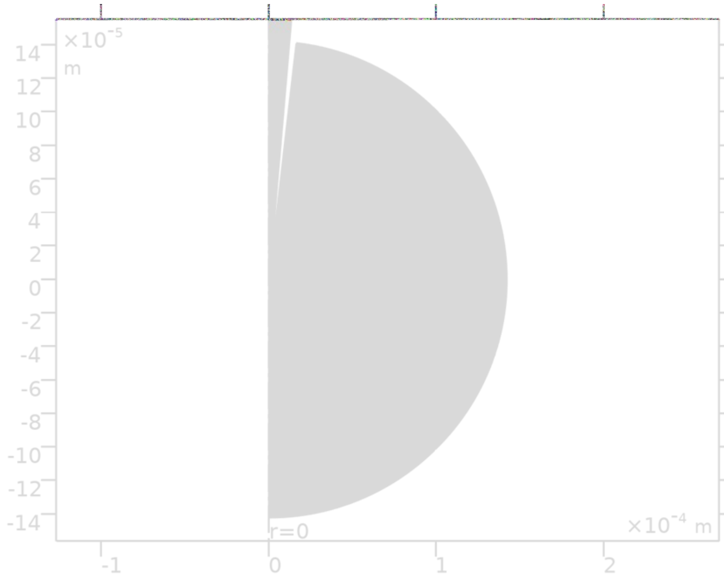

Initial Values (Gouy-Chapman in Bath)

SELECTION

Geometric entity level	Domain
Selection	Geometry geom1: Dimension 2: Domains 1–2, 4

SETTINGS

**Description**	**Value**
Electric potential	Potential_GCBath + AnalyticalPotentialNoCharge

2.4.8 Space Charge Density

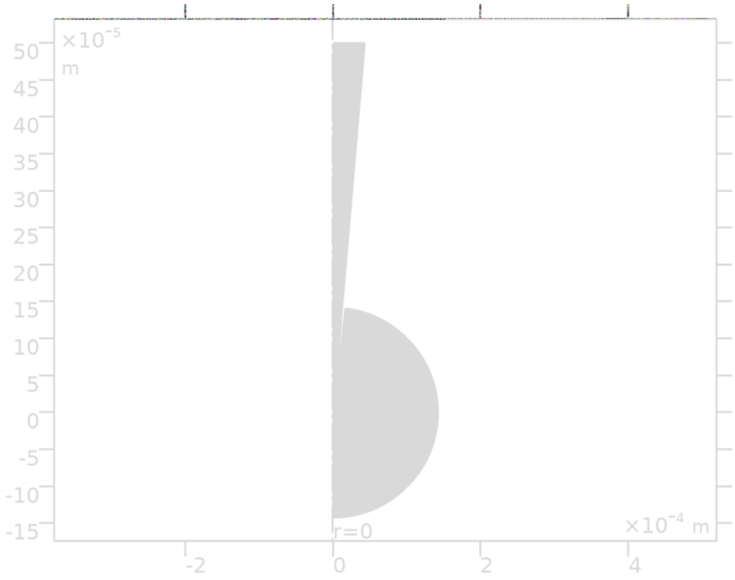

Space Charge Density

SELECTION

Geometric entity level	Domain
Selection	Geometry geom1: Dimension 2: Domains 1–4

EQUATIONS

$$\nabla \cdot \mathbf{D} = \rho_v$$

Coordinate System Selection

SETTINGS

Description	Value
Coordinate system	Global coordinate system

2.4.9 Surface Charge (Glass Wall)

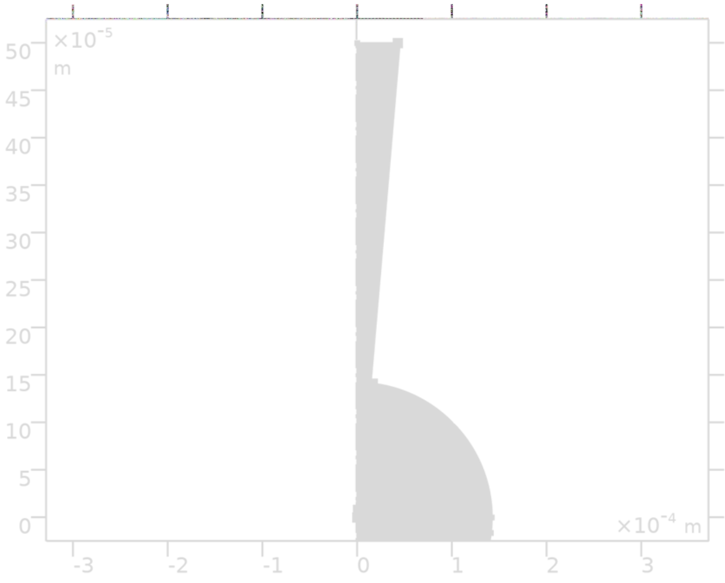

Surface Charge (Glass Wall)

SELECTION

Geometric entity level	Boundary
Name	Glass Wall
Selection	Named sel1: Geometry geom1: Dimension 1: Boundaries 7–9, 11

EQUATIONS

$$\mathbf{n} \cdot (\mathbf{D}_1 - \mathbf{D}_2) = \rho_s$$

Surface Charge Density

SETTINGS

Description	Value
Surface charge density	PoreSurfaceCharge

Coordinate System Selection

SETTINGS

Description	Value
Coordinate system	Global coordinate system

2.4.10 Surface Charge (Au nanoparticle)

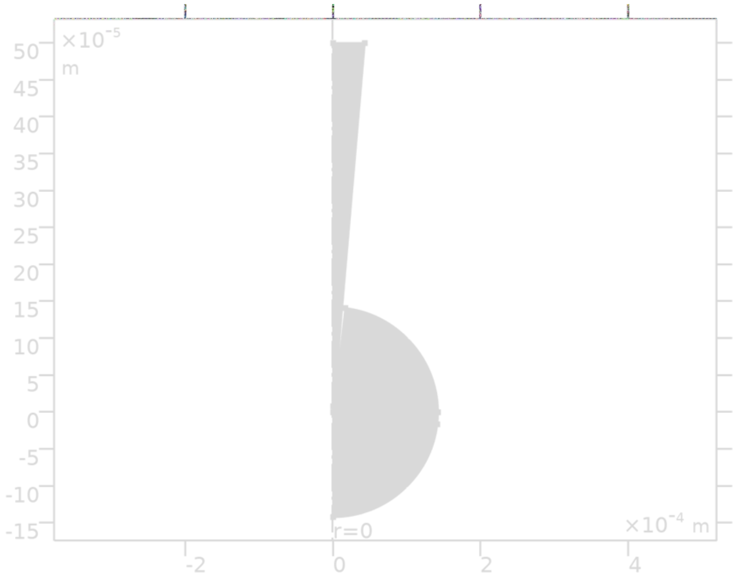

Surface Charge (Au nanoparticle)

SELECTION

Geometric entity level	Boundary
Selection	Geometry geom1: Dimension 1: No boundaries

EQUATIONS

$$\mathbf{n}\cdot(\mathbf{D}_1 - \mathbf{D}_2) = \rho_s$$

Surface Charge Density

SETTINGS

**Description**	**Value**
Surface charge density	ParticleSurfaceCharge

Coordinate System Selection

SETTINGS

**Description**	**Value**
Coordinate system	Global coordinate system

2.4.11 Electric Potential (Gouy-Chapman, external electrode)

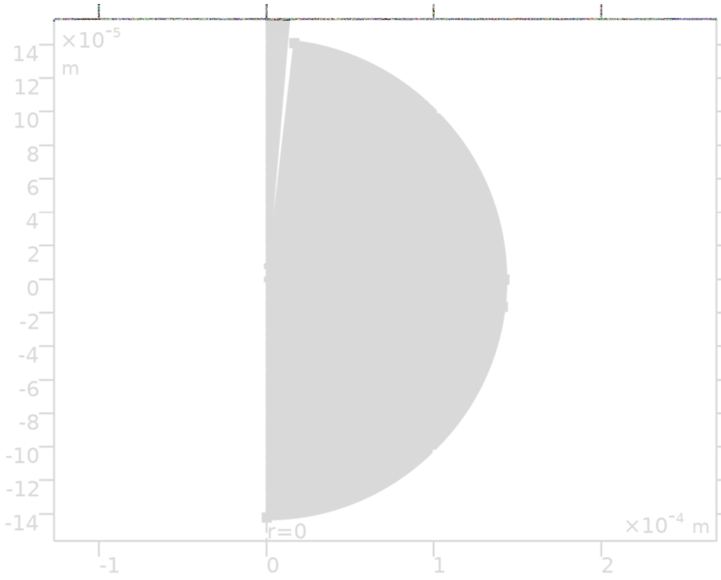

Electric Potential (Gouy-Chapman, external electrode)

SELECTION

Geometric entity level	Boundary
Name	External Electrode
Selection	Named sel4: Geometry geom1: Dimension 1: Boundaries 12–14

EQUATIONS

$$V = V_0$$

Electric Potential

SETTINGS

Description	Value
Electric potential	E_GCBath(dGlass)

2.4.12 Electric Potential (Gouy-Chapman, internal electrode)

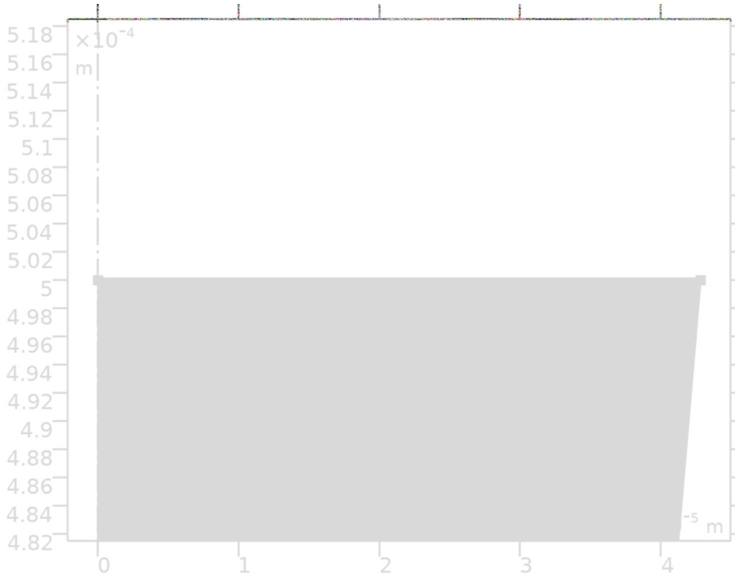

Electric Potential (Gouy-Chapman, internal electrode)

SELECTION

Geometric entity level	Boundary
Name	Internal Electrode
Selection	Named sel3: Geometry geom1: Dimension 1: Boundary 6

EQUATIONS

$$V = V_0$$

Electric Potential

SETTINGS

**Description**	**Value**
Electric potential	VApp + E_GC(dGlass)

2.5 FINAL MESH

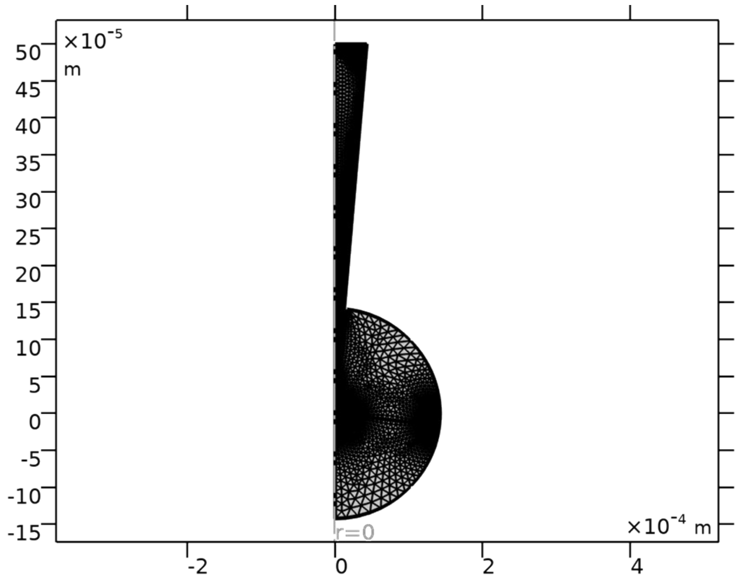

Final Mesh

2.5.1 Size Overall (size)

SETTINGS

**Description**	**Value**
Maximum element size	200E-7
Minimum element size	5E-10
Curvature factor	0.2
Resolution of narrow regions	10
Predefined size	Extremely fine
Custom element size	Custom

2.5.2 Size for Glass Wall (size2)

SELECTION

Geometric entity level	Boundary
Name	Glass Wall
Selection	Named sel1: Geometry geom1: Dimension 1: Boundaries 7–9, 11

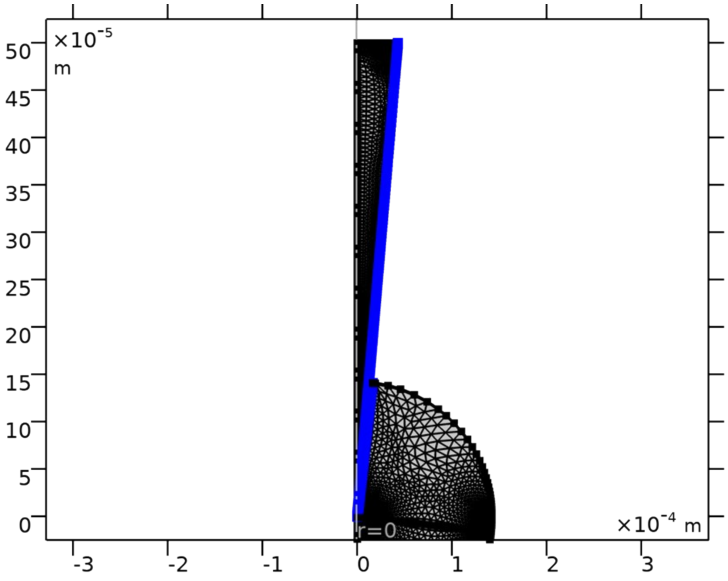

Size for Glass Wall

SETTINGS

Description	Value
Maximum element size	6.42E-6
Minimum element size	1.28E-8
Curvature factor	0.2
Predefined size	Extremely fine

2.5.3 Size Aperture Points (size1)

SELECTION

Geometric entity level	Point
Selection	Geometry geom1: Dimension 0: Points 5–6, 9

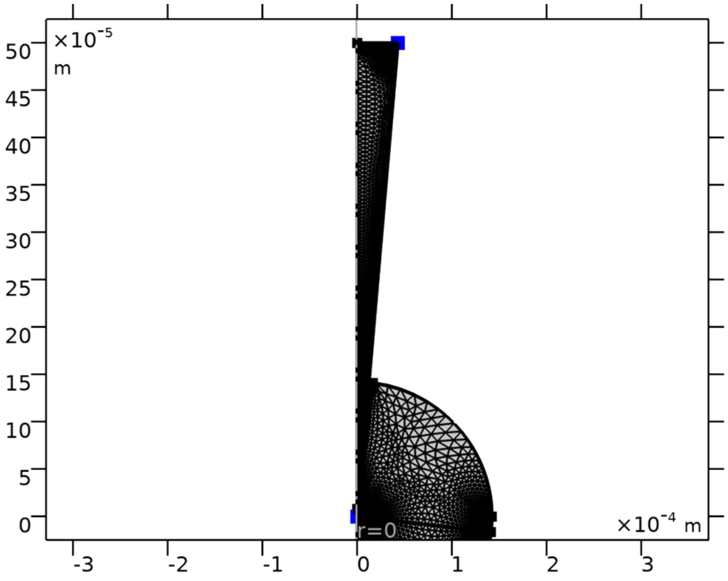

Size Aperture Points

SETTINGS

Description	Value
Maximum element size	PoreRadius/100
Minimum element size	6.45E-10
Minimum element size	Off
Curvature factor	0.3
Curvature factor	Off
Resolution of narrow regions	Off
Maximum element growth rate	1.3
Maximum element growth rate	Off
Custom element size	Custom

2.5.4 Boundary Layers (bl1)

SELECTION

Geometric entity level	Domain
Selection	Geometry geom1

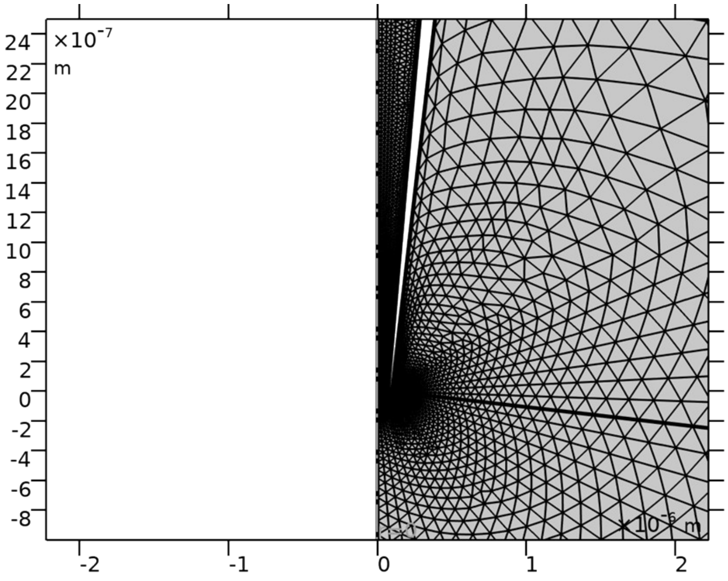

Boundary Layers

SETTINGS

Description	Value
Number of iterations	30
Maximum element depth to process	30
Last build time	7
Built with	COMSOL 6.0.0.354 (glnxa64)|2024 - 06 - 06T16:50:16.266986

Boundary Layer Properties (Internal) (blp)

SELECTION

Geometric entity level	Boundary
Selection	Geometry geom1: Dimension 1: Boundaries 8, 11

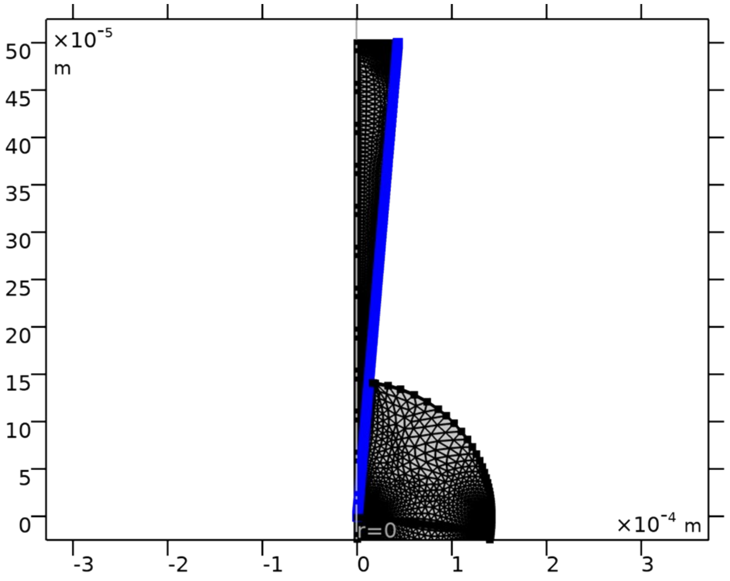

Boundary Layer Properties (Internal)

SETTINGS

Description	Value
Number of layers	12
Thickness specification	First layer
Thickness	DebyeLengthPore/10

Boundary Layer Properties (External) (blp1)

SELECTION

Geometric entity level	Boundary
Selection	Geometry geom1: Dimension 1: Boundaries 7, 9

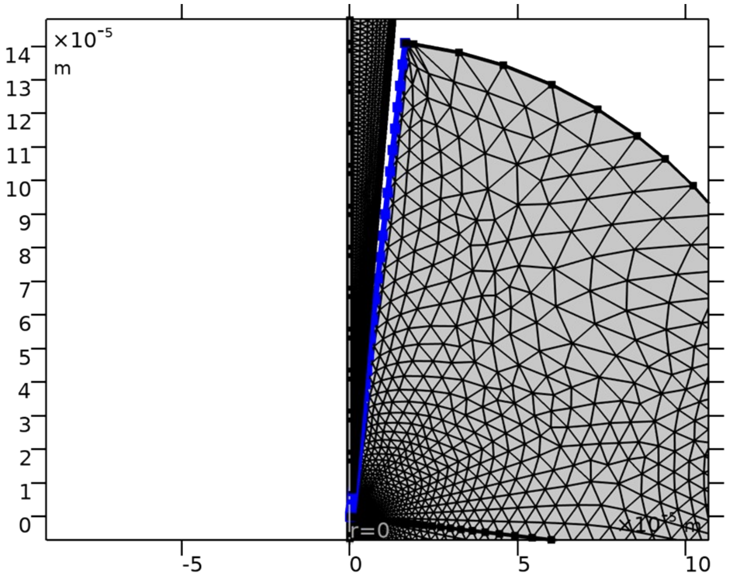

Boundary Layer Properties (External)

SETTINGS

Description	Value
Number of layers	12
Thickness specification	First layer
Thickness	DebyeLengthBath/10

3 Steady State

COMPUTATION INFORMATION

Computation time	42 s

3.1 STATIONARY

STUDY SETTINGS

Description	Value
Include geometric nonlinearity	Off

PHYSICS AND VARIABLES SELECTION

Physics interface	Discretization
Transport of Diluted Species (chds)	physics
Electrostatics (es)	physics

MESH SELECTION

Geometry	Mesh
Nanopipette (geom1)	mesh2

3.2 SOLVER CONFIGURATIONS

3.2.1 Solution 1

Compile Equations: Stationary (st1)

STUDY AND STEP

Description	Value
Use study	Steady State
Use study step	Stationary

Dependent Variables 1 (v1)

GENERAL

Description	Value
Defined by study step	Stationary

Concentration (mod1.cCl) (mod1_cCl)

GENERAL

Description	Value
Field components	mod1.cCl
Internal variables	{mod1.uflux.cCl, mod1.dflux.cCl}

Concentration (mod1.cK) (mod1_cK)

GENERAL

Description	Value
Field components	mod1.cK
Internal variables	{mod1.uflux.cK, mod1.dflux.cK}

Electric potential (mod1.V) (mod1_V)

GENERAL

Description	Value
Field components	mod1.V

Stationary Solver 1 (s1)

GENERAL

Description	Value
Defined by study step	Stationary

RESULTS WHILE SOLVING

Description	Value
Plot	On
Plot group	Probe Plot Group 1

Advanced (aDef)

ASSEMBLY SETTINGS

Description	Value
Reuse sparsity pattern	On

Fully Coupled 1 (fc1)

GENERAL

Description	Value
Linear solver	Direct, concentrations (chds) (merged)

METHOD AND TERMINATION

Description	Value
Initial damping factor	0.01
Minimum damping factor	1.0E-6
Maximum number of iterations	50

4 Results

4.1 DATA SETS

4.1.1 Steady State/Solution 1

SOLUTION

Description	Value
Solution	Solution 1
Component	Model 1 (mod1)

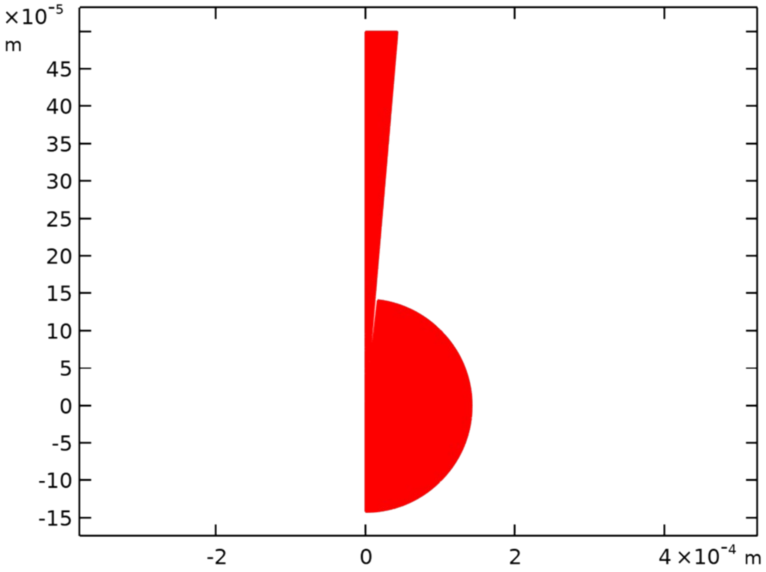

Dataset: Steady State/Solution 1

4.1.2 Probe Solution 2

SOLUTION

Description	Value
Solution	Solution 1
Component	Model 1 (mod1)

Dataset: Probe Solution 2

4.1.3 Current (internal electrode)

SELECTION

Geometric entity level	Boundary
Selection	Geometry geom1: Dimension 1: Boundary 6

DATA

Description	Value
Dataset	Probe Solution 2

SETTINGS

Description	Value
Method	Integration
Integration order	4
Integration order	On

4.2 DERIVED VALUES

4.2.1 Current (internal electrode)

OUTPUT

Evaluated in	Probe Table 1

DATA

Description	Value
Dataset	Current (internal electrode)

EXPRESSIONS

Expression	Unit	Description
-F_const*(chds.bndFlux_cK-chds.bndFlux_cCl)*2*pi*r	nA	Current (internal)
